# Hemorrhage-coagulation immune phenotype is associated with CD163/HO-1-enriched heme-processing macrophage remodeling and predicts recurrence in colorectal cancer: a real-world retrospective cohort study

**DOI:** 10.3389/fimmu.2026.1868145

**Published:** 2026-06-29

**Authors:** Yiqi Yan, Siyu Mao

**Affiliations:** Department of Clinical Laboratory, The First People’s Hospital of Jiashan County, Jiaxing, China

**Keywords:** coagulation, colorectal cancer, fibrinogen, hemorrhage, immunohistochemistry, macrophage, tumor microenvironment

## Abstract

**Background:**

Coagulation activation and tumor hemorrhage are common in colorectal cancer, but their combined immunological meaning remains incompletely defined. We developed a prespecified hemorrhage-coagulation immune phenotype (HCIP) integrating local hemorrhagic pathology and systemic coagulation activation, and evaluated its association with recurrence-free survival (RFS), CD163/HO-1-enriched immune remodeling, and incremental prognostic value.

**Methods:**

This retrospective cohort study included patients who underwent radical resection for colorectal adenocarcinoma at The First People’s Hospital of Jiashan County between January 2016 and December 2025. HCIP was defined from a local hemorrhagic axis, based on H&E-assessed intratumoral hemorrhage and hemosiderin/Prussian blue evidence, and a systemic coagulation axis, based on preoperative fibrinogen, D-dimer, and platelet count. The primary outcome was RFS. A balanced FFPE subset was used for exploratory immunohistochemical assessment of CD8, CD68, CD163, HO-1, fibrinogen/fibrin, and tissue factor. Cox models, prespecified sensitivity analyses, and validation-cohort prediction analyses with bootstrap uncertainty estimates were performed.

**Results:**

The final analytic cohort included 752 patients. HCIP-poor tumors were enriched for advanced AJCC stage, larger tumor size, ulceration, and necrosis. In the balanced FFPE subset, HCIP-poor tumors showed lower CD8+ stromal T-cell density, higher CD163+ macrophage density, stronger HO-1, fibrinogen/fibrin, and tissue factor staining, a lower CD8/CD163 ratio, and a higher macrophage-heme-coagulation score. In multivariable Cox analysis, HCIP-poor status was associated with worse RFS (adjusted HR 1.91, 95% CI 1.33-2.73). The association remained directionally consistent in stage II/III-only, T/N-stage-adjusted, MSI/MMR-stratified, IPTW, and recurrence-only sensitivity analyses. Among individual hemorrhage/coagulation components, fibrinogen was the most stable marker associated with RFS. In the validation cohort, adding fibrinogen, HCIP, or hemorrhage/coagulation components produced modest incremental discrimination and lower prediction error beyond clinical variables.

**Conclusion:**

HCIP identifies a clinically accessible hemorrhage-coagulation state associated with CD163/HO-1-enriched heme-processing macrophage features and coagulation-associated immune remodeling in colorectal cancer. Fibrinogen remained the most practical single coagulation marker, whereas HCIP provided a biologically interpretable composite phenotype linking local hemorrhagic pathology, systemic coagulation activation, and recurrence risk.

## Introduction

Colorectal cancer remains one of the major causes of cancer incidence and cancer-related mortality worldwide, and recurrence after curative-intent surgery continues to define a clinically important subgroup of patients who need better risk stratification (1, 2). Conventional clinicopathological variables, particularly tumor stage, lymphovascular invasion, perineural invasion, and treatment history, remain indispensable, but they do not fully capture the biological heterogeneity that underlies postoperative recurrence.

A large body of work has shown that the immune contexture of colorectal cancer has prognostic value beyond anatomy-based staging. Early studies demonstrated that the type, density, and location of immune cells in colorectal tumors were strongly associated with clinical outcome (3). The international validation of the consensus Immunoscore further supported the clinical relevance of T-cell-based immune assessment in colon cancer, with recurrence prediction that complements TNM staging (4). These studies established the principle that a histologically accessible immune phenotype can carry clinically meaningful information.

At the same time, the immune microenvironment is not shaped by lymphocytes alone. Tumor-associated macrophages are abundant stromal cells that can support invasion, angiogenesis, matrix remodeling, and suppression of adaptive immunity (5, 6). In colorectal cancer, macrophage markers such as CD163 and macrophage balance indices have been linked to prognosis and immune organization, suggesting that macrophage-centered states may modify the meaning of T-cell infiltration (7, 8). This is particularly relevant in tumors with hemorrhagic or necrotic stroma, where macrophages are exposed not only to tumor-derived cytokines but also to erythrocyte breakdown products.

Coagulation is another underappreciated feature of the tumor microenvironment. Cancer can activate coagulation at both systemic and intratumoral levels, and coagulation products can in turn regulate inflammation, immune cell recruitment, angiogenesis, and metastasis (9, 10). Pretreatment fibrinogen and D-dimer have been associated with poor outcomes in several digestive cancers, including colorectal cancer (11, 12). Tissue factor and fibrinogen/fibrin deposition also provide a local route through which tumor-associated procoagulant activity may influence stromal architecture and tumor cell dissemination (13–15).

Hemorrhage may represent a missing link between coagulation activation and macrophage-centered immune remodeling. CD163 is a hemoglobin-haptoglobin scavenger receptor, and the CD163-HO-1 pathway participates in hemoglobin clearance and heme metabolism in macrophages (16, 17). In tissues exposed to erythrocyte leakage and heme, macrophages can acquire programs related to heme processing, iron handling, and immune regulation (18). Whether local hemorrhagic pathology and systemic coagulation activation jointly identify a recurrence-relevant immune phenotype in colorectal cancer has not been well characterized.

We therefore developed a prespecified hemorrhage-coagulation immune phenotype (HCIP), integrating a local hemorrhagic axis with a systemic coagulation axis. Using a real-world tertiary-hospital cohort from The First People’s Hospital of Jiashan County, we evaluated whether HCIP was associated with RFS after radical resection, whether it corresponded to CD163/HO-1 macrophage and coagulation-related IHC features, and whether it added prognostic information beyond conventional clinicopathological variables.

## Materials and methods

### Study design and population

This retrospective cohort study was conducted at The First People’s Hospital of Jiashan County, Jiaxing, Zhejiang, China. A total of 1,138 patients were screened from the radical colorectal adenocarcinoma surgery registry between January 2016 and December 2025. Patients were eligible if they had analyzable postoperative follow-up, available baseline clinicopathological data, and preoperative routine coagulation and blood count measurements. Patients were excluded for stage IV disease or non-radical surgery (n=96), neoadjuvant treatment before the index surgery (n=114), synchronous malignancy or other ineligible history (n=35), unavailable key hemorrhage/coagulation/pathology variables (n=72), or unavailable RFS data or inadequate follow-up (n=69), leaving 752 patients in the final analytic cohort. The study was approved by the ethics committee of The First People’s Hospital of Jiashan County (approval number: js02332). Because this was a retrospective study using de-identified clinical data and archived specimens, the requirement for individual informed consent was waived according to institutional policy.

### Definition of the hemorrhage-coagulation immune phenotype

HCIP was defined before outcome modeling as a rule-based composite phenotype integrating a local hemorrhagic axis and a systemic coagulation activation axis. The definition was designed to capture the coexistence of pathological hemorrhagic features and systemic coagulation activation, rather than to maximize survival discrimination.

For the local hemorrhagic axis, H&E-stained whole-section slides were reviewed for intratumoral hemorrhage in viable tumor and tumor-stromal regions. Intratumoral hemorrhage was scored on a four-tier scale: 0, no evident red blood cell extravasation; 1, scattered or focal red blood cell extravasation involving only a small stromal area; 2, definite multifocal hemorrhage or a localized hemorrhagic stromal area; and 3, extensive or confluent hemorrhage involving broad tumor-stromal regions. Hemosiderin deposition was assessed using Prussian blue staining when available or pathological evidence of hemosiderin-laden macrophages. Hemosiderin was scored as 0, absent; 1, rare scattered granules; 2, definite focal granular deposits; and 3, multifocal or abundant granular deposits. The local hemorrhagic axis was considered positive when the H&E hemorrhage score was ≥2 or the hemosiderin score was ≥2.

For the systemic coagulation axis, preoperative fibrinogen, D-dimer, and platelet count were used because they are routinely available and biologically related to coagulation activation. Fibrinogen >4.0 g/L, D-dimer >1.0 mg/L, and platelet count ≥300 × 10^9/L were considered abnormal. The systemic coagulation axis was considered positive when at least two of these three abnormalities were present. Patients were classified as HCIP-favorable when both axes were negative, HCIP-intermediate when either the local hemorrhagic axis or the systemic coagulation axis was positive, and HCIP-poor when both axes were positive (**Supplementary Table 1**).

Pathological hemorrhage and hemosiderin scoring were performed independently by two observers who were blinded to recurrence status and laboratory results. Discrepant scores were resolved by joint review and consensus. The HCIP classification was generated after pathological and laboratory variables had been finalized and was not optimized according to recurrence-free survival.

### Outcome definition

The primary outcome was recurrence-free survival, defined as the interval from radical surgery to first documented local recurrence, distant metastasis, or disease-specific death when documented, whichever occurred first. Disease-specific death was determined from the medical record when death was attributed to colorectal cancer progression or recurrence. Patients without an event were censored at the date of the last documented follow-up. Deaths without documented recurrence or disease-specific attribution were not counted as RFS events and were censored at the death date when available. A recurrence-only sensitivity analysis was performed in which death without documented recurrence was treated as censoring.

### Clinical, pathological, and laboratory data

Demographic variables, tumor location, AJCC stage, histological grade, tumor size, lymphovascular invasion, perineural invasion, MMR/MSI status, tumor ulceration, tumor necrosis, CEA status, and postoperative treatment information were extracted from the electronic medical record and pathology reporting system. Preoperative laboratory values were obtained from routine tests closest to surgery and before major perioperative intervention. Coagulation-related variables included fibrinogen, D-dimer, and platelet count. Systemic inflammation-related indices, including the systemic immune-inflammation index (SII), were calculated from routine blood count data when available. Preoperative laboratory values were defined as the closest available measurements within 14 days before surgery and before any major perioperative transfusion, anticoagulation adjustment, or emergency intervention when such information was available. For the HCIP definition, fibrinogen >4.0 g/L and D-dimer >1.0 mg/L were considered abnormal according to commonly used institutional reference limits, and platelet count ≥300 × 10^9/L was considered elevated. These thresholds were selected before outcome modeling to preserve clinical interpretability and avoid survival-optimized cutoff selection.

### Immunohistochemical validation subset

IHC analyses were performed in a balanced FFPE subset rather than the entire cohort because the purpose of the IHC component was exploratory assessment of HCIP-associated immune and coagulation-related features. Approximately 30 specimens were selected from each HCIP group among cases with available high-quality FFPE tumor blocks, adequate viable tumor tissue, evaluable stromal area, and complete clinicopathological and follow-up information. The subset was selected to ensure representation across HCIP phenotypes and was not intended to estimate marker prevalence in the full cohort. Therefore, IHC-based analyses were interpreted as phenotype-level biological assessment rather than proof of a causal immunological mechanism.

For each selected case, representative tumor blocks containing invasive tumor and adjacent tumor stroma were retrieved. Necrotic areas, extensive cautery artifact, mucin pools without viable tumor, and areas with poor fixation were avoided for quantitative assessment. When multiple eligible blocks were available, the block containing the most representative tumor-stromal interface was selected.

### Immunohistochemistry and image quantification

Four-micrometer-thick sections were cut from FFPE tumor blocks and mounted on adhesive glass slides. Sections were deparaffinized in xylene, rehydrated through graded ethanol, and subjected to heat-induced antigen retrieval using citrate buffer or EDTA buffer according to the manufacturer’s recommendations for each antibody. Endogenous peroxidase activity was blocked with hydrogen peroxide, followed by incubation with primary antibodies against CD8, CD68, CD163, HO-1, fibrinogen/fibrin, and tissue factor. Detection was performed using a horseradish peroxidase-conjugated secondary antibody system and 3,3′-diaminobenzidine chromogen, followed by hematoxylin counterstaining. Appropriate positive and negative controls were included in each staining batch.

CD8, CD68, and CD163 were quantified in stromal regions as positive cell density. For each specimen, representative stromal fields within the invasive tumor region were selected while avoiding necrosis, tissue folds, and major hemorrhagic pools without viable tumor. Positive cells were counted in multiple high-power fields and converted to cells/mm². HO-1, fibrinogen/fibrin, and tissue factor staining were evaluated using an H-score approach based on staining intensity and the proportion of positive cells or stromal area. Staining intensity was graded as 0, negative; 1, weak; 2, moderate; and 3, strong. H-score was calculated as intensity × percentage of positive area or cells, yielding a score from 0 to 300. For fibrinogen/fibrin and tissue factor, H-score assessment focused on viable invasive tumor-stromal regions and adjacent stromal or perivascular staining while avoiding necrotic debris, tissue folds, and large hemorrhagic pools without viable tumor. Staining localization was recorded descriptively to distinguish stromal/perivascular deposition from nonspecific necrosis-associated staining.

The CD8/CD163 ratio was calculated as stromal CD8^+^ cell density divided by stromal CD163^+^ cell density. The exploratory macrophage–heme–coagulation score was calculated as the mean of standardized CD163 density, HO-1 H-score, fibrinogen/fibrin H-score, and tissue factor H-score. A higher score indicated coordinated enrichment of CD163^+^ macrophages, heme-processing activity, and local coagulation-associated protein deposition. This composite score was used only for mechanistic interpretation in the IHC subset and was not included in full-cohort prediction models.

IHC assessment was performed by observers blinded to HCIP group and recurrence outcome. Disagreements in representative field selection or scoring were resolved by consensus review. When digital image analysis was available, quantitative measurements were performed using standardized color deconvolution and thresholding settings across batches; otherwise, manual counting and semiquantitative scoring were performed using the same field-selection rules.

### Prediction modeling

Prediction analyses were performed to evaluate the incremental value of hemorrhage–coagulation features beyond conventional clinicopathological variables. The full analytic cohort was split into training and validation cohorts using a stratified 7:3 approach, with stratification by RFS event status and HCIP group. Model development and tuning were performed exclusively in the training cohort, and final performance was evaluated in the validation cohort. The final split yielded 525 patients in the model-development cohort and 227 patients in the validation cohort.

The reference clinical model included age, sex, body mass index, tumor location, AJCC stage, histological grade, tumor size, lymphovascular invasion, perineural invasion, tumor deposit, MMR/MSI status, tumor ulceration, tumor necrosis, albumin, elevated CEA, adjuvant chemotherapy, and postoperative radiotherapy. IHC variables were not included in full-cohort prediction models because they were measured only in the balanced FFPE subset and were intended for exploratory tissue-level assessment. Incremental feature sets included clinical variables plus fibrinogen, clinical variables plus HCIP, and clinical variables plus individual hemorrhage/coagulation components. HCIP and its component variables were not forced into the same primary incremental Cox model.

Continuous variables were retained on their clinical scale for Cox models. For penalized Cox and random survival forest models, categorical variables were dummy-coded, and continuous variables were centered and scaled using parameters estimated in the training cohort and then applied to the validation cohort. Missing values were imputed using training-cohort medians for continuous variables and the most frequent category or an explicit missing category for categorical variables. LASSO-Cox and elastic-net Cox models were fitted using penalized partial likelihood with cross-validation for tuning parameter selection (19, 20). Random survival forest was fitted as a non-parametric robustness model (21). Validation performance was assessed using Harrell’s C-index and time-dependent AUCs at 36 and 60 months (22). Incremental performance was summarized relative to the clinical Cox model. Uncertainty around validation performance was quantified using 500 bootstrap resamples of the validation cohort. Prediction error was assessed using Brier scores, and clinical utility was explored using 36-month decision-curve analysis. Calibration was summarized by validation-cohort risk quintiles.

### Statistical analysis

Continuous variables were summarized as medians and interquartile ranges, and categorical variables as counts and percentages. Differences across HCIP groups were assessed using Kruskal-Wallis tests for continuous variables and chi-square tests for categorical variables. Kaplan-Meier curves and log-rank tests were used to compare RFS across HCIP groups. Multivariable Cox proportional hazards models estimated adjusted hazard ratios and 95% confidence intervals. Models 1 and 2 were adjusted for age, sex, tumor location, AJCC stage, histological grade, lymphovascular invasion, perineural invasion, elevated CEA, and adjuvant chemotherapy. The exploratory IHC Cox model was adjusted for age, sex, AJCC stage, lymphovascular invasion, and perineural invasion. The covariate set used for inferential Cox models differed from the broader prediction feature set because the former was intended for adjusted association estimation, whereas the latter was intended for validation-cohort risk prediction. HCIP and downstream IHC mechanistic markers were not forced into the same primary model to avoid overadjustment and suppression effects. Because the IHC subset was balanced by HCIP group and was not a random sample of the full cohort, IHC comparisons were interpreted as phenotype-level tissue assessment rather than full-cohort prevalence estimates. The exploratory IHC Cox model was reported to support biological interpretation and was not used for model development. Prediction performance was evaluated using validation-cohort C-index and time-dependent AUCs at 36 and 60 months (22). Sensitivity analyses were performed using alternative HCIP definitions, including stricter hemorrhagic and coagulation thresholds, an H&E-only hemorrhagic axis combined with fibrinogen/D-dimer, a percentile-based composite definition, and exclusion of patients receiving antithrombotic therapy. Reporting was guided by principles of transparent prediction model reporting (23). Additional sensitivity and diagnostic analyses included missing-data summaries, Schoenfeld residual-based proportional hazards tests, restricted cubic spline checks for fibrinogen, D-dimer, platelet count, SII, age, tumor size, and albumin, stage II/III-only Cox models, separate T-stage and N-stage adjustment, MSI/MMR-stratified models, stabilized inverse-probability-weighted analyses, feature-set ablation analyses, recurrence-only endpoint analysis, bootstrap confidence intervals for incremental discrimination metrics, Brier scores, calibration summaries, and decision-curve analysis.

## Results

### Cohort construction and baseline characteristics

**Figure 1:**
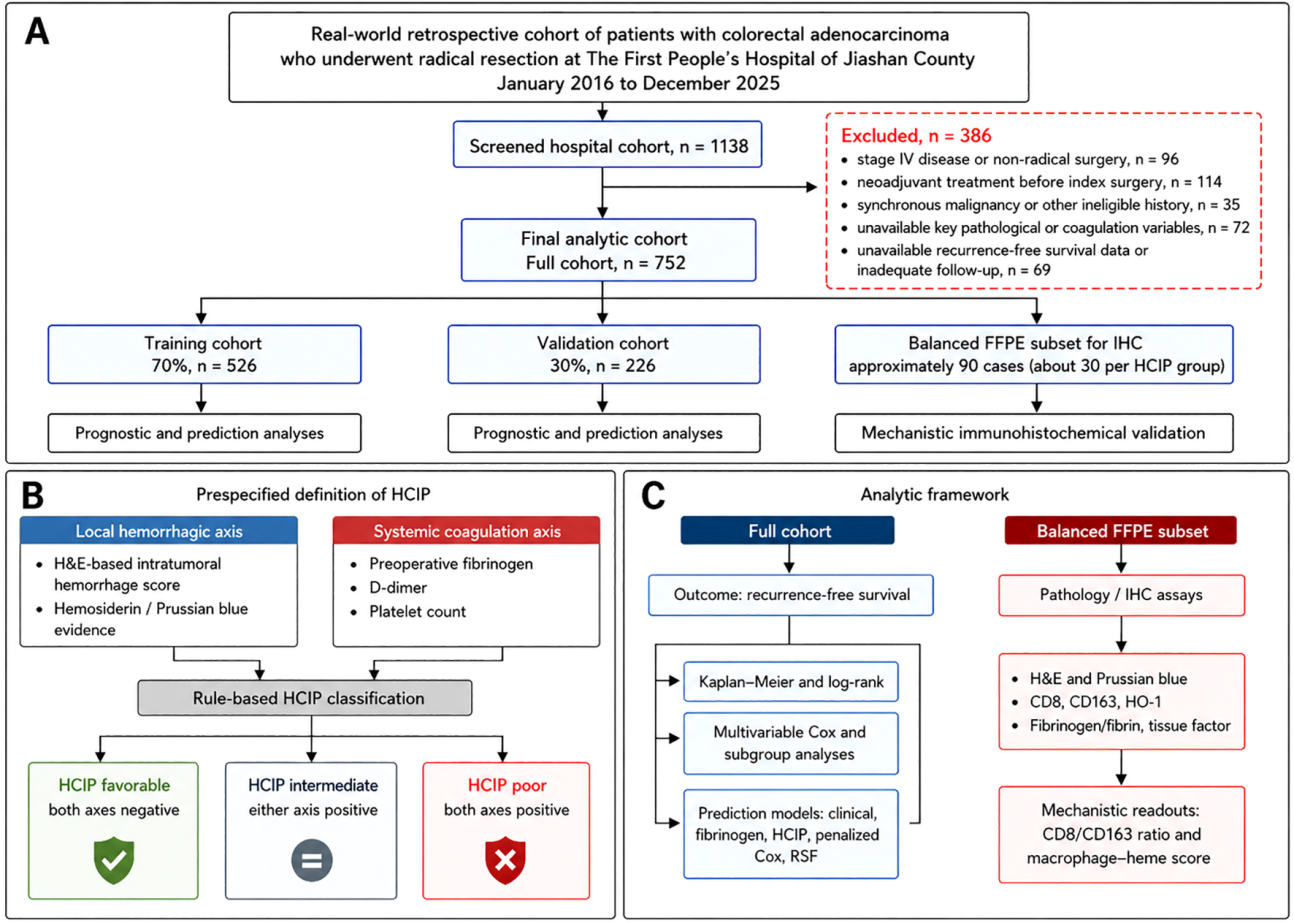
Study design, cohort construction, and definition of the hemorrhage-coagulation immune phenotype. **(A)** Flow diagram of the retrospective tertiary-hospital cohort. A total of 1,138 patients who underwent radical resection for colorectal adenocarcinoma between January 2016 and December 2025 were screened. After exclusion of patients with stage IV disease or non-radical surgery, neoadjuvant treatment, synchronous malignancy or other ineligible history, unavailable key hemorrhage/coagulation/pathology variables, unavailable recurrence-free survival data, or inadequate follow-up, 752 patients remained in the final analytic cohort. A stratified 7:3 split generated model-development and validation cohorts. A balanced FFPE subset was selected for exploratory IHC assessment. **(B)** Prespecified definition of HCIP. The local hemorrhagic axis was based on H&E-assessed intratumoral hemorrhage and hemosiderin/Prussian blue evidence, whereas the systemic coagulation axis was based on preoperative fibrinogen, D-dimer, and platelet count. Patients were classified as HCIPfavorable when both axes were negative, HCIP-intermediate when either axis was positive, and HCIP-poor when both axes were positive. **(C)** Analytic framework. The full cohort was used for recurrence-free survival analysis, multivariable Cox and subgroup analyses, and prediction modeling. The balanced FFPE subset was used for exploratory pathology/IHC assays, including H&E, Prussian blue, CD8, CD163, HO-1, fibrinogen/fibrin, and tissue factor, and for evaluation of mechanistic readouts including the CD8/CD163 ratio and macrophage–heme–coagulation score.

Among 1,138 screened patients, 386 were excluded according to prespecified criteria, leaving 752 patients with resected colorectal adenocarcinoma in the final analytic cohort (**Figure 1**; **Supplementary Tables 3**, **4**). The model-development and validation cohorts included 525 and 227 patients, respectively. Overall reverse Kaplan-Meier median follow-up was 42.9 months (95% CI 37.7-46.4), and the observed median follow-up time was 29.6 months (interquartile range 13.5-54.6). According to the prespecified HCIP definition, 312 patients were classified as HCIP-favorable, 280 as HCIP-intermediate, and 160 as HCIP-poor. Median age in the overall cohort was 62.0 years, and 451 patients (60.0%) were men. Tumor location was distributed across the right colon (28.5%), left colon (31.4%), and rectum (40.2%). AJCC stage differed across HCIP groups: stage III disease was present in 36.5% of HCIP-favorable, 50.0% of HCIP-intermediate, and 73.8% of HCIP-poor tumors (P<0.001). Tumor size also increased across HCIP groups, with a median of 4.6 cm in HCIP-favorable tumors and 5.3 cm in HCIP-poor tumors (P<0.001). Tumor ulceration and necrosis were more frequent in the HCIP-poor group. In contrast, sex, body mass index, tumor location, histological grade, lymphovascular invasion, perineural invasion, MMR/MSI status, elevated CEA, and adjuvant treatment distributions were not materially different across HCIP groups (**Table 1**; **Supplementary Tables 5**, **6**).

**Table 1:** Clinicopathological characteristics according to HCIP.

Characteristic	Overall N = 752	Favorable N = 312	Intermediate N = 280	Poor N = 160	P value
Age, years	62.0 (55.0, 69.0)	64.0 (57.0, 69.5)	60.5 (54.0, 68.0)	61.5 (53.5, 68.0)	0.008
Female sex	301 (40.0%)	130 (41.7%)	100 (35.7%)	71 (44.4%)	0.151
Body mass index, kg/m2	23.2 (21.5, 25.5)	23.2 (21.6, 25.5)	23.3 (21.5, 25.4)	23.1 (21.5, 25.7)	0.920
Tumor location: right/left/rectum	214/236/302	88/97/127	78/99/103	48/40/72	0.247
AJCC stage I/II/III	110/270/372	75/123/114	31/109/140	4/38/118	<0.001
Histological grade well/moderate/poor	72/552/128	32/231/49	27/208/45	13/113/34	0.581
Tumor size, cm	4.9 (3.8, 6.1)	4.6 (3.6, 5.8)	4.8 (3.8, 6.2)	5.3 (4.3, 6.5)	<0.001
Lymphovascular invasion	252 (33.5%)	104 (33.3%)	87 (31.1%)	61 (38.1%)	0.320
Perineural invasion	188 (25.0%)	75 (24.0%)	74 (26.4%)	39 (24.4%)	0.782
MSI-H/dMMR	92 (12.2%)	38 (12.2%)	34 (12.1%)	20 (12.5%)	0.993
Tumor ulceration	412 (54.8%)	123 (39.4%)	168 (60.0%)	121 (75.6%)	<0.001
Tumor necrosis	314 (41.8%)	94 (30.1%)	118 (42.1%)	102 (63.8%)	<0.001
Elevated CEA	512 (68.1%)	203 (65.1%)	194 (69.3%)	115 (71.9%)	0.279
Adjuvant chemotherapy	401 (53.3%)	161 (51.6%)	151 (53.9%)	89 (55.6%)	0.686
Postoperative radiotherapy	141 (18.8%)	59 (18.9%)	54 (19.3%)	28 (17.5%)	0.895

Values are median (interquartile range) or n (%), unless otherwise indicated. P values were calculated using Kruskal-Wallis tests for continuous variables and chi-square tests for categorical variables. HCIP-defining hemorrhage and coagulation variables are summarized in the **Supplementary Material**. AJCC, American Joint Committee on Cancer; CEA, carcinoembryonic antigen; HCIP, hemorrhage-coagulation immune phenotype; MMR, mismatch repair; MSI, microsatellite instability.

### Immunohistochemical immune microenvironment across HCIP groups

**Figure 2:**
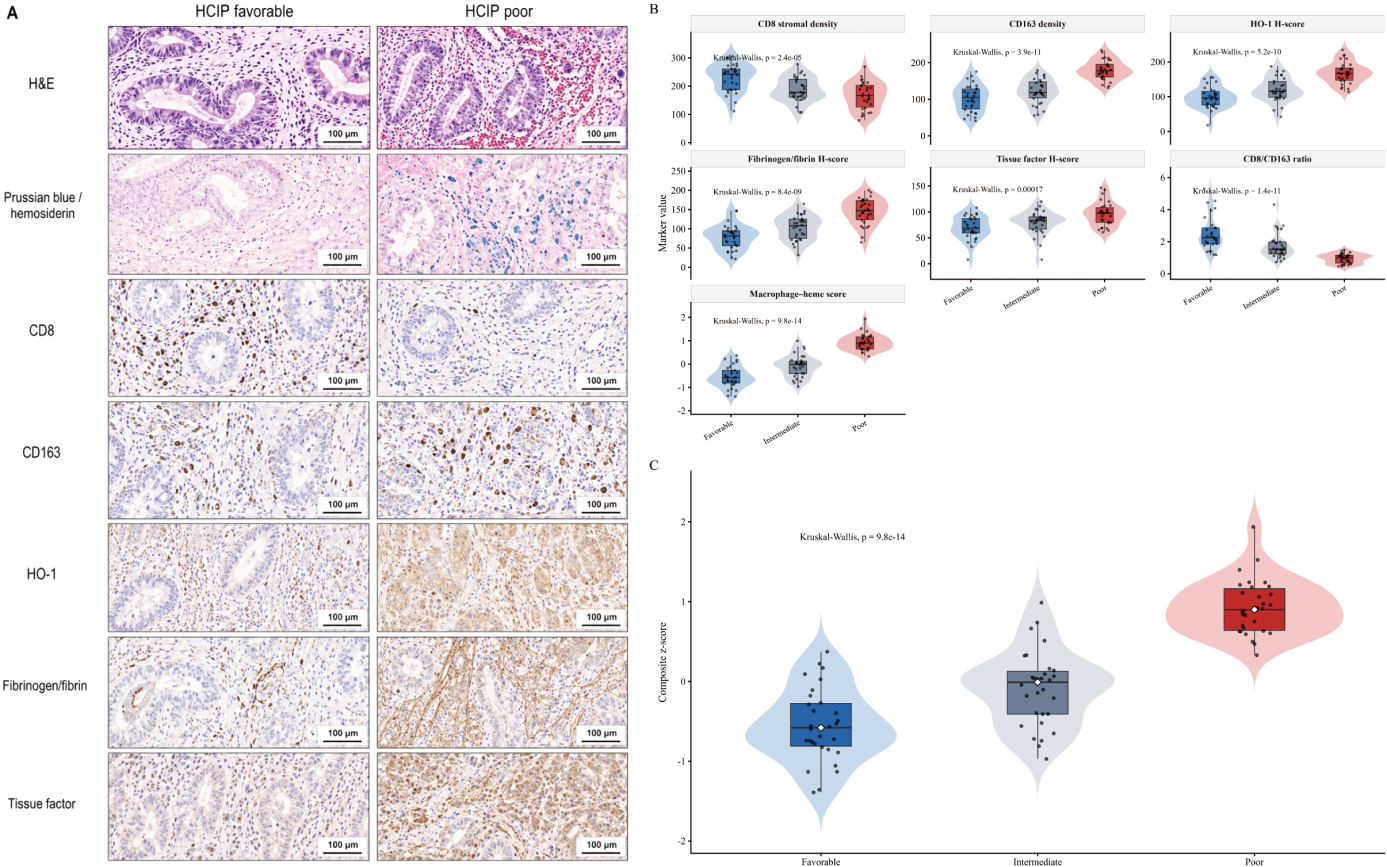
Representative pathology and immunohistochemical features according to HCIP. **(A)** Representative H&E, Prussian blue/hemosiderin, and IHC images comparing HCIP-favorable and HCIP-poor tumors. HCIP-favorable tumors showed relatively preserved glandular architecture, minimal intratumoral hemorrhage, sparse hemosiderin deposition, and more abundant CD8+ stromal lymphocytes. HCIP-poor tumors showed more prominent stromal hemorrhage and red blood cell extravasation, granular hemosiderin deposition, reduced CD8+ lymphocyte infiltration, increased CD163+ macrophage infiltration, stronger HO-1 expression, and greater fibrinogen/fibrin and tissue factor staining. Scale bars, 100 micrometers. **(B)** Quantitative IHC features across HCIP groups in the balanced FFPE subset. **(C)** Macrophage–heme–coagulation score across HCIP groups. The score was calculated as the mean of standardized CD163 density, HO-1 H-score, fibrinogen/fibrin H-score, and tissue factor H-score. Group comparisons were performed using the Kruskal-Wallis test. Fibrinogen/fibrin and tissue factor staining were interpreted primarily in viable tumor-stromal and perivascular regions, with necrotic debris and nonrepresentative hemorrhagic pools avoided for scoring.

IHC analysis was performed in a balanced FFPE subset of 90 cases, with 30 specimens selected from each HCIP group. HCIP-poor tumors had a lower CD8+ stromal T-cell density than HCIP-favorable tumors (median 166.9 versus 242.4; P<0.001). CD163+ macrophage density increased markedly across HCIP groups, with the highest density in the HCIP-poor group (median 178.1; P<0.001). HO-1 H-score, fibrinogen H-score, and tissue factor H-score also increased across HCIP groups (all P<0.001). CD68+ macrophage density showed a more modest pattern and did not differ significantly across groups (P = 0.201), suggesting that the phenotype was more closely aligned with CD163/HO-1-associated macrophage features than with total macrophage abundance alone. The CD8/CD163 ratio decreased from 2.3 in HCIP-favorable tumors to 1.0 in HCIP-poor tumors (P<0.001), and the macrophage–heme–coagulation score increased from -0.6 to 0.9 (P<0.001) (**Table 2**; **Figure 2**). Fibrinogen/fibrin and tissue factor staining were evaluated primarily in viable tumor-stromal and perivascular compartments; scoring avoided necrotic debris and nonrepresentative hemorrhagic pools. Descriptive MSI/MMR-stratified IHC summaries are provided in **Supplementary Table 17**, but the IHC subset was not powered for formal MSI-stratified inference. Descriptively, fibrinogen/fibrin staining in HCIP-poor tumors was most prominent in stromal/perivascular regions adjacent to viable tumor, and tissue factor staining was distributed in both tumor-cell-associated and adjacent stromal regions, rather than being confined to necrotic debris.

**Table 2:** Immunohistochemical features in the balanced FFPE-IHC subset.

IHC marker	Overall N = 90	Favorable N = 30	Intermediate N = 30	Poor N = 30	P value
CD8+ stromal T-cell density	191.9 (160.3, 237.9)	242.4 (187.7, 261.1)	178.0 (160.3, 224.6)	166.9 (122.6, 204.1)	<0.001
CD68+ macrophage density	179.7 (152.9, 203.5)	170.3 (146.9, 192.4)	180.4 (152.1, 203.5)	182.8 (168.1, 216.1)	0.201
CD163+ macrophage density	135.6 (102.2, 170.0)	105.2 (71.4, 128.5)	118.5 (102.2, 148.3)	178.1 (160.4, 196.6)	<0.001
HO-1 H-score	123.0 (97.5, 161.9)	97.0 (75.7, 116.9)	115.5 (97.5, 145.0)	167.0 (145.9, 182.6)	<0.001
Fibrinogen H-score	106.4 (77.7, 140.7)	81.8 (55.8, 95.3)	108.0 (73.5, 126.0)	147.5 (122.2, 173.5)	<0.001
Tissue factor H-score	83.9 (64.1, 97.1)	68.7 (59.3, 87.4)	83.5 (65.9, 90.6)	97.7 (79.4, 109.4)	<0.001
CD8/CD163 ratio	1.4 (1.1, 2.1)	2.3 (1.9, 2.9)	1.5 (1.2, 2.0)	1.0 (0.7, 1.2)	<0.001
Macrophage–heme–coagulation score	0.0 (-0.6, 0.7)	-0.6 (-0.8, -0.3)	0.0 (-0.4, 0.1)	0.9 (0.6, 1.2)	<0.001

The IHC analysis was performed in a balanced FFPE-IHC subset selected at approximately 30 specimens per HCIP group (actual N = 90). Values are median (interquartile range). P values were calculated using Kruskal-Wallis tests. The macrophage–heme–coagulation score was calculated from standardized CD163, HO-1, fibrinogen, and tissue factor measurements.

### HCIP and recurrence-free survival

**Figure 3:**
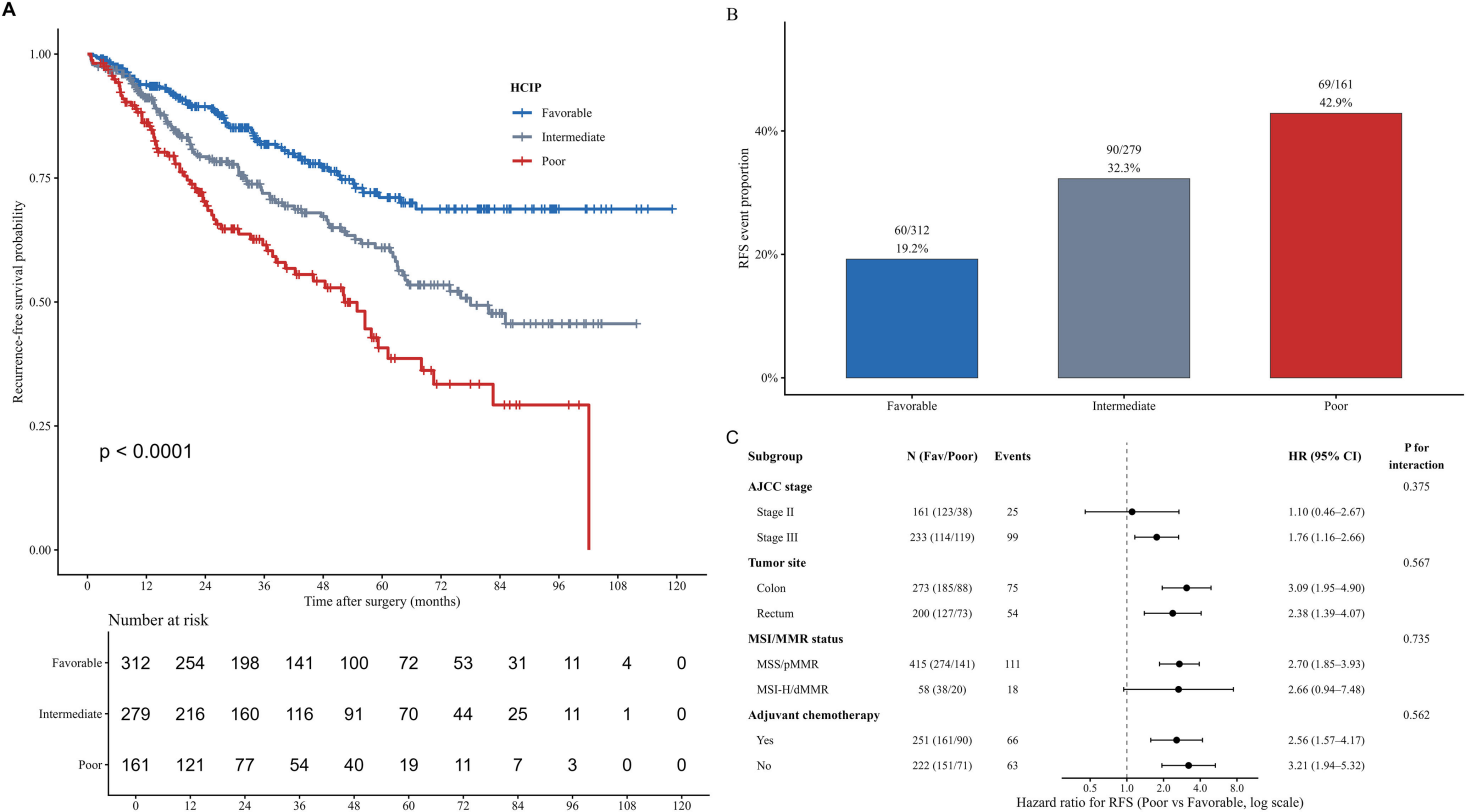
Recurrence-free survival according to hemorrhage-coagulation immune phenotype. **(A)** Kaplan-Meier curves for RFS according to HCIP group. Patients with HCIP-poor tumors showed the lowest RFS probability, whereas patients with HCIP-favorable tumors showed the most favorable RFS profile. The log-rank test compared survival distributions across groups, and the risk table shows the number at risk over time. **(B)** RFS event proportions across HCIP groups. Event rates increased from HCIP-favorable to HCIP-intermediate and HCIP-poor groups. **(C)** Subgroup analysis of the association between HCIP-poor status and RFS. Hazard ratios compare HCIP-poor with HCIP-favorable tumors within clinically relevant subgroups. Models were adjusted for age and sex when estimable. P values for interaction assessed whether the association differed across subgroup strata.

Kaplan-Meier analysis showed clear separation of RFS curves across HCIP groups, with the lowest RFS probability in the HCIP-poor group (**Figure 3A**). The proportion of RFS events increased across HCIP groups (**Figure 3B**). In multivariable Cox analysis adjusted for clinicopathological covariates and adjuvant chemotherapy, HCIP-poor status was associated with worse RFS compared with HCIP-favorable status (adjusted HR 1.91, 95% CI 1.33-2.73, P<0.001). HCIP-intermediate status showed a directionally higher risk but did not reach conventional statistical significance (adjusted HR 1.36, 95% CI 0.97-1.90, P = 0.074). Subgroup analyses were consistent in direction, without evidence that the association differed materially by AJCC stage, tumor site, MMR/MSI status, or adjuvant chemotherapy status (**Figure 3C**).

### Individual hemorrhage/coagulation components and exploratory IHC Cox analysis

When individual hemorrhage and coagulation variables were evaluated in a multivariable Cox model without HCIP, fibrinogen was the most stable individual marker associated with RFS (adjusted HR 1.90 per 1 g/L increase, 95% CI 1.48-2.45, P<0.001). H&E hemorrhage score, hemosiderin score, D-dimer, platelet count, and SII did not show statistically significant independent associations in the same model, although SII showed a borderline trend (adjusted HR 1.16 per 500-unit increase, 95% CI 0.99-1.36, P = 0.071). In the exploratory IHC subset model, the macrophage–heme–coagulation score was associated with worse RFS (adjusted HR 2.18 per SD increase, 95% CI 1.23-3.89, P = 0.008), whereas the CD8/CD163 ratio was not statistically significant after adjustment (**Table 3**).

**Table 3:** Core multivariable associations with recurrence-free survival.

Model	Analysis set	Variable	Adjusted HR (95% CI)	P value
Model 1: HCIP phenotype	Full cohort; n=752; events=219	HCIP intermediate vs favorable	1.36 (0.97-1.90)	0.074
		HCIP poor vs favorable	1.91 (1.33-2.73)	<0.001
Model 2: Hemorrhage/coagulation components	Full cohort; n=752; events=219	H&E hemorrhage score, per 1-grade increase	0.91 (0.78-1.06)	0.209
		Hemosiderin score, per 1-grade increase	1.00 (0.86-1.16)	0.987
		Fibrinogen, per 1 g/L increase	1.90 (1.48-2.45)	<0.001
		D-dimer, per 1 mg/L increase	1.11 (0.94-1.33)	0.226
		Platelet count, per 50 x10^9/L increase	0.97 (0.84-1.12)	0.640
		SII, per 500-unit increase	1.16 (0.99-1.36)	0.071
Model 3: IHC mechanistic markers	Exploratory IHC subset; n=90	CD8/CD163 ratio, per SD increase	0.87 (0.47-1.61)	0.662
		Macrophage–heme–coagulation score, per SD increase	2.18 (1.23-3.89)	0.008

Only hypothesis-relevant variables are shown. Models 1 and 2 were fitted in the full analytic cohort. Model 3 was fitted in the balanced IHC subset and should be interpreted as exploratory mechanistic analysis. Models 1 and 2 were adjusted for age, sex, tumor location, AJCC stage, histological grade, lymphovascular invasion, perineural invasion, elevated CEA, and adjuvant chemotherapy. Model 3 was adjusted for age, sex, AJCC stage, lymphovascular invasion, and perineural invasion. HCIP and downstream IHC mechanistic markers were not forced into the same main model to avoid overadjustment and suppression effects. HR, hazard ratio; CI, confidence interval; SII, systemic immune-inflammation index.

### Incremental prediction performance

**Figure 4:**
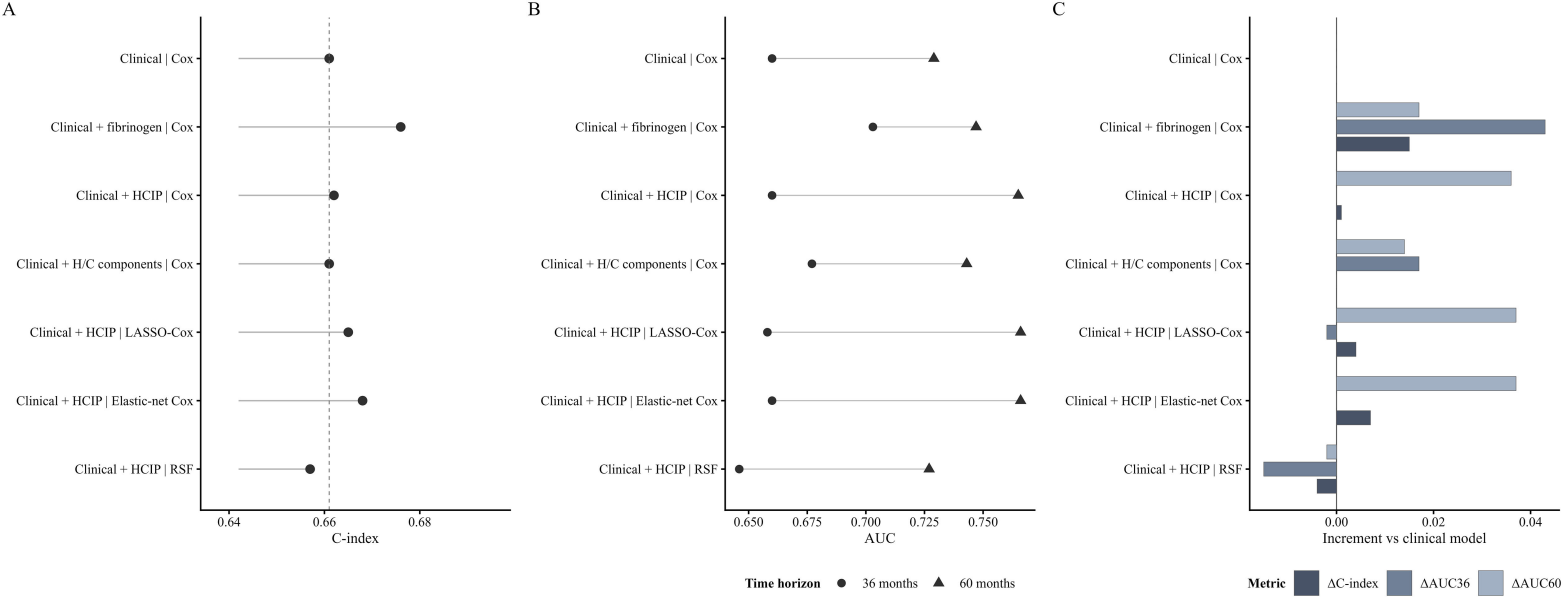
Incremental validation performance of hemorrhage-coagulation features for recurrence-free survival prediction. **(A)** Validation C-index values with 95% bootstrap confidence intervals. **(B)** Time-dependent AUCs at 36 and 60 months with 95% bootstrap confidence intervals. **(C)** Incremental changes in C-index, 36-month AUC, and 60-month AUC compared with the clinical Cox model. The clinical Cox model was used as the reference, and incremental models evaluated the added value of fibrinogen, HCIP, and hemorrhage/coagulation components beyond conventional clinicopathological variables. IHC variables were not included in fullcohort prediction models because they were measured only in the balanced FFPE subset. Brier scores are reported in **Table 4**, and decision-curve/calibration analyses are provided in the **Supplementary Material**.

In the independent validation cohort, the clinical Cox model achieved a C-index of 0.676 (95% bootstrap CI 0.600-0.742), with time-dependent AUCs of 0.756 (95% CI 0.662-0.829) at 36 months and 0.743 (95% CI 0.652-0.835) at 60 months (**Supplementary Figure 2**; **Supplementary Table 15**). Adding fibrinogen increased the C-index to 0.696, adding HCIP increased the C-index to 0.686, and adding individual hemorrhage/coagulation components increased the C-index to 0.695. The corresponding 36-month AUCs were 0.768, 0.753, and 0.768, respectively. Brier scores were lower for the incremental models than for the clinical reference model (**Table 4**). Bootstrap confidence intervals for delta C-index and time-dependent AUC generally crossed zero, supporting a conservative interpretation of modest incremental discrimination (**Supplementary Table 16**). Decision-curve and calibration analyses are shown in **Figure 4** and the **Supplementary Material** (**Supplementary Figures 3**, **4**; **Supplementary Table 14**). These results suggest that hemorrhage-coagulation features provide modest incremental information beyond clinical variables, with fibrinogen emerging as the most practical single coagulation marker and HCIP serving as a biologically interpretable composite phenotype.

**Table 4:** Incremental performance of hemorrhage-coagulation features for recurrence-free survival prediction in the validation cohort.

Model	C-index (95% CI)	36-month AUC (95% CI)	60-month AUC (95% CI)	36-month Brier score (95% CI)	60-month Brier score (95% CI)
Clinical model	0.676 (0.600-0.742)	0.756 (0.662-0.829)	0.743 (0.652-0.835)	0.276 (0.228-0.324)	0.326 (0.260-0.391)
Clinical + fibrinogen	0.696 (0.616-0.763)	0.768 (0.680-0.838)	0.779 (0.691-0.859)	0.214 (0.165-0.264)	0.261 (0.211-0.320)
Clinical + HCIP	0.686 (0.608-0.746)	0.753 (0.661-0.830)	0.769 (0.682-0.847)	0.214 (0.176-0.253)	0.251 (0.192-0.312)
Clinical + hemorrhage/coagulation components	0.695 (0.615-0.762)	0.768 (0.675-0.841)	0.788 (0.702-0.868)	0.210 (0.162-0.257)	0.250 (0.201-0.303)

The clinical Cox model served as the reference. Incremental models evaluated fibrinogen, HCIP, and hemorrhage/coagulation components beyond clinical variables. Values are shown as estimates with 95% bootstrap confidence intervals from validation-cohort resampling. Lower Brier scores indicate lower prediction error. AUC, area under the curve; HCIP, hemorrhage-coagulation immune phenotype.

### Sensitivity analyses

Sensitivity analyses supported the direction of the main findings. Under the main definition, HCIP-poor status was associated with worse RFS (HR 1.91, 95% CI 1.33-2.73). The association remained directionally consistent using alternative HCIP definitions and after exclusion of antithrombotic users (**Supplementary Table 2**). In additional models requested for residual confounding assessment, HCIP-poor remained associated with RFS in stage II/III-only analyses (HR 1.95, 95% CI 1.29-2.94), in models adjusting for T stage and N stage separately (HR 2.09, 95% CI 1.39-3.13), and in stage II/III-only models with separate T/N-stage adjustment (HR 1.93, 95% CI 1.28-2.91) (**Supplementary Table 9**). A Cox model stratified by MSI/MMR status gave a similar estimate for HCIP-poor status (HR 2.00, 95% CI 1.33-3.01), and recurrence-only sensitivity analysis also remained consistent (HR 2.06, 95% CI 1.38-3.10) (**Supplementary Tables 10**, **11**). Stabilized IPTW analyses yielded directionally supportive results, although selected tumor-burden variables retained partial residual imbalance after weighting (**Supplementary Tables 12**, **13**). Proportional hazards testing did not suggest violation for HCIP or the global model; MSI/MMR status showed evidence of non-proportionality and was addressed through MSI-stratified sensitivity analysis (**Supplementary Table 7**). Restricted cubic spline analyses did not show statistically significant nonlinearity for fibrinogen, D-dimer, platelet count, SII, age, tumor size, or albumin (**Supplementary Figure 1**; **Supplementary Table 8**). , .

## Discussion

This study proposes and evaluates HCIP as a prespecified, clinically accessible phenotype that integrates local hemorrhagic pathology and systemic coagulation activation in resected colorectal cancer. Three observations are central. First, HCIP-poor tumors were associated with worse RFS after adjustment for established clinicopathological factors and remained directionally consistent across stage-, MSI/MMR-, IPTW-, and recurrence-only sensitivity analyses. Second, in a balanced FFPE subset, HCIP-poor tumors showed a coherent immune pattern: fewer CD8+ stromal T cells, more CD163+ macrophages, stronger HO-1 expression, greater fibrinogen/fibrin and tissue factor staining, and a lower CD8/CD163 ratio. Third, fibrinogen emerged as the most stable individual coagulation marker, whereas HCIP provided an integrative framework linking coagulation activation with hemorrhagic pathology and CD163/HO-1-enriched heme-processing macrophage features.

The value of HCIP should not be interpreted as simple competition with fibrinogen. In our data, fibrinogen was the most parsimonious single coagulation marker and provided the clearest short-term incremental signal. This aligns with previous evidence that elevated pretreatment fibrinogen is associated with adverse outcomes in CRC and other cancers (11, 12, 24). Fibrinogen is attractive clinically because it is inexpensive, routinely measured, and analytically stable. However, fibrinogen alone reflects systemic coagulation activation; it does not capture local hemorrhage, hemosiderin deposition, or the immune consequences of heme exposure. HCIP was therefore designed not as a replacement for fibrinogen, but as a composite phenotype that adds histopathological context to the coagulation signal. The validation analyses reinforced this interpretation: HCIP did not clearly outperform fibrinogen as a standalone prognostic tool, but it contextualized systemic coagulation activation within a local hemorrhagic and immune-pathological phenotype.

This distinction is important for biological interpretation. The coagulation system interacts with cancer through more than thromboembolism. Coagulation proteins and fibrin deposits can shape tumor stroma, support tumor cell adhesion, and modulate inflammatory and immune processes (9, 10, 25). Tissue factor can initiate extrinsic coagulation and also signal through protease-activated pathways that affect angiogenesis and immune regulation (13, 15). Fibrinogen/fibrin deposition in the tumor bed may therefore act not only as a marker of systemic inflammation or clotting, but also as a local scaffold for stromal remodeling and immune cell organization (26). The increased fibrinogen/fibrin and tissue factor staining seen in HCIP-poor tumors is consistent with this concept.

**Figure 5:**
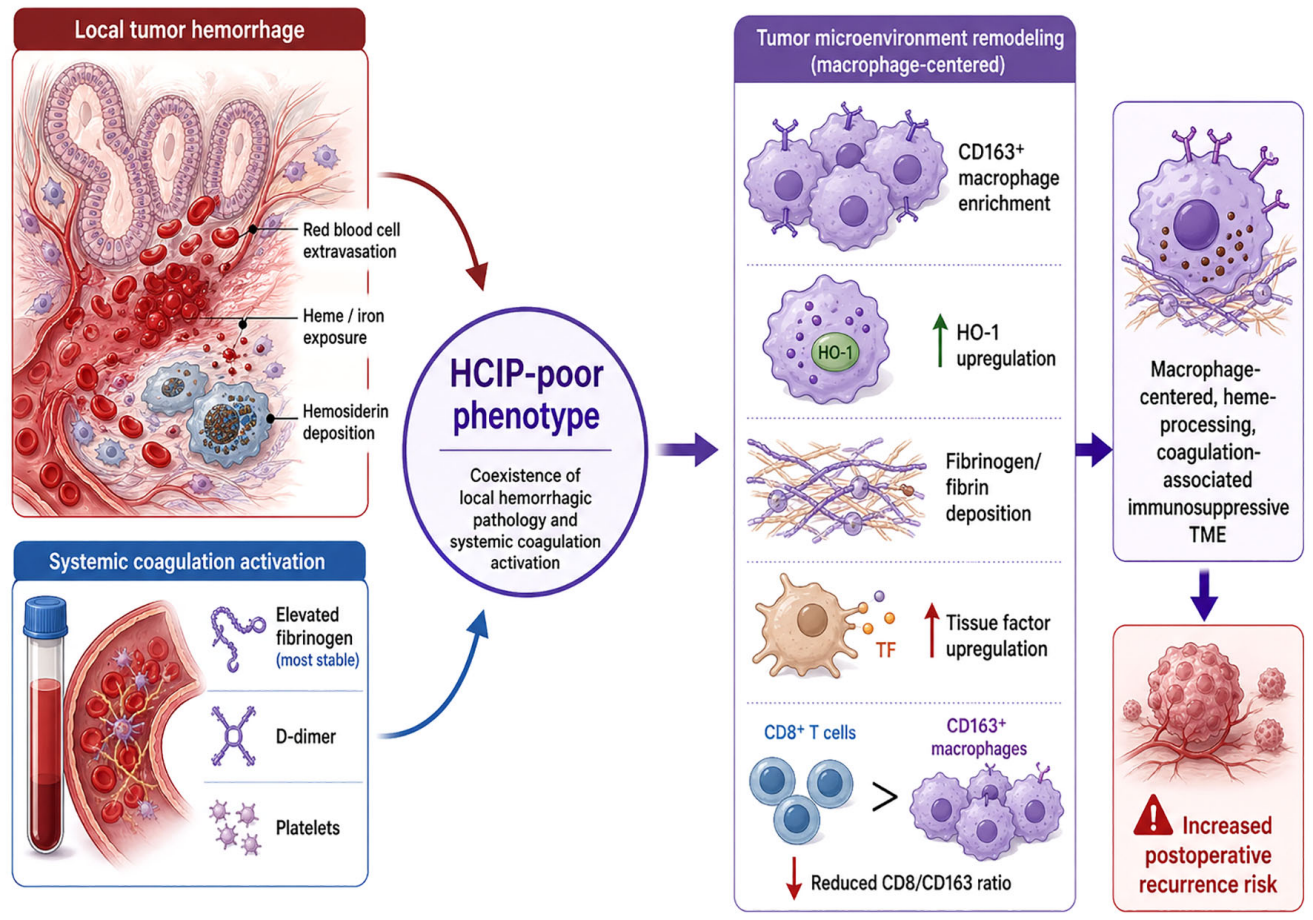
Hypothesis-generating model linking HCIP to CD163/HO-1-enriched heme-processing macrophage features and recurrence risk. Local tumor hemorrhage and hemosiderin/iron deposition may reflect repeated red blood cell extravasation and heme exposure within the tumor microenvironment. In parallel, systemic coagulation activation, particularly elevated fibrinogen, may contribute to local fibrinogen/fibrin deposition and tissue factor-related procoagulant activity. The coexistence of local hemorrhagic pathology and systemic coagulation activation defines the HCIP-poor phenotype. This phenotype is associated with enrichment of CD163+/HO-1+ macrophage-related features, increased fibrinogen/fibrin and tissue factor staining, and a reduced CD8/CD163 ratio. The schematic should be interpreted as a hypothesis-generating biological model rather than a demonstrated causal mechanism.

The macrophage findings provide a biologically plausible link between hemorrhagic tumor pathology and macrophage-associated immune remodeling. CD163 is a hemoglobin–haptoglobin scavenger receptor, and the CD163–HO-1 axis participates in hemoglobin clearance and heme metabolism in macrophages (16, 17). Erythrocyte extravasation and heme exposure is consistent with formation of a local niche in which macrophages may acquire iron-handling and tissue-repair programs (18). In tumors, such programs may coexist with angiogenesis, matrix remodeling, and altered adaptive immune organization rather than representing a single macrophage activation state. The higher macrophage–heme–coagulation score in HCIP-poor tumors supports the view that hemorrhagic and coagulation-rich tumors may be enriched for a CD163/HO-1-centered heme-processing macrophage pattern (**Figure 5**). Accordingly, the present data should be interpreted as showing a CD163/HO-1-enriched heme-processing macrophage pattern rather than proving a specific M2-like or functionally suppressive macrophage state.

This finding fits with the broader role of macrophages in cancer. TAMs are highly plastic and can support tumor progression by remodeling matrix, promoting vascular programs, suppressing cytotoxic immunity, and shaping response to therapy (5, 6, 27). In colorectal cancer, macrophage markers and macrophage balance have been associated with prognosis, although the direction and magnitude of associations depend on location, phenotype, and interaction with T-cell infiltration (7, 28, 29). Our data suggest that CD163 enrichment is most informative when interpreted together with CD8 depletion and heme/coagulation markers, rather than as a single isolated macrophage count.

The reduction in CD8/CD163 ratio across HCIP groups is also biologically meaningful. Colorectal cancer was among the first tumors in which the prognostic value of immune infiltration was systematically demonstrated (3). The Immunoscore later showed that quantification of CD3/CD8 cells in tumor regions can stratify recurrence risk beyond classical staging (4). Our study does not attempt to reproduce the Immunoscore. Instead, it asks a different question: whether hemorrhagic and coagulation features identify a stromal state in which T-cell infiltration is counterbalanced or constrained by macrophage-centered remodeling. The lower CD8/CD163 ratio in HCIP-poor tumors is consistent with this interpretation. Because multiplex or spatially registered image analysis was not available, this study assessed immune-cell density and selected compartmental staining rather than formal spatial immune exclusion or CD8-macrophage proximity.

A related consideration is how HCIP relates to the canonical MSI/MMR-driven immune phenotype of colorectal cancer. MSI-H/dMMR tumors typically display a high mutational burden, increased neoantigen load, and a CD8-enriched, inflamed tumor immune microenvironment, which represents a well-established axis of immunological heterogeneity in colorectal cancer. HCIP is not intended to replace this classical stratification; rather, it captures a complementary dimension centered on local hemorrhagic pathology, systemic coagulation activation, and CD163/HO-1-enriched heme-processing macrophage features, which is biologically distinct from antigenicity-driven T-cell infiltration. Consistent with this interpretation, HCIP-poor status remained directionally associated with worse RFS within the MSS/pMMR subgroup in our MSI/MMR-stratified sensitivity analyses, suggesting that HCIP captures prognostic information beyond MSI-driven immune inflammation, although the MSI-H/dMMR subgroup was too small to support definitive within-stratum inference.

The prediction analyses should be read conservatively. Adding fibrinogen, HCIP, or hemorrhage/coagulation components to clinical models produced only modest improvements in validation-cohort discrimination. Penalized Cox models provided slightly higher C-indices than the clinical reference model, whereas random survival forest did not improve performance. This pattern suggests that the added value was feature-driven rather than algorithm-driven. It also reinforces a common observation in clinical prediction: complex algorithms do not necessarily outperform well-specified conventional models, particularly when the underlying predictors carry moderate effects and sample size is limited (23, 30). Bootstrap confidence intervals for incremental discrimination metrics further support a cautious interpretation, because most delta C-index and delta AUC intervals included zero, whereas Brier score reductions suggested lower prediction error for several incremental feature sets. Calibration plots showed that incremental models including fibrinogen tended to compress predicted risks toward the low end of the probability scale in the validation cohort, indicating that further recalibration in independent external cohorts will be required before any clinical use.

A strength of this study is the integration of routinely available clinical pathology, preoperative coagulation tests, and targeted IHC validation. The HCIP definition was prespecified and not optimized to maximize a survival statistic. Its association with RFS remained directionally consistent across sensitivity analyses, including alternative definitions and exclusion of antithrombotic users. The IHC subset was deliberately balanced across HCIP groups, which strengthens phenotype-level comparisons, but it should not be interpreted as estimating full-cohort marker prevalence. For practical implementation, HCIP can be scored using routine H&E review for intratumoral hemorrhage, Prussian blue or documented hemosiderin-laden macrophages when available, and preoperative fibrinogen, D-dimer, and platelet count obtained before major perioperative intervention. Discordant local and systemic results are classified as HCIP-intermediate by design.

Several limitations deserve emphasis. First, the study was retrospective and single-center, which limits causal inference and external generalizability. Second, treatment decisions and follow-up intensity may have varied over the 2016–2025 period, despite adjustment for major clinicopathological and treatment variables. Third, hemorrhage and hemosiderin scoring may be affected by block selection and regional tumor heterogeneity. Fourth, the IHC subset was designed for exploratory phenotype-level assessment, not full-cohort biomarker modeling; therefore, the IHC Cox analysis should be considered exploratory. Fifth, this study measured immune-cell density and selected staining compartments rather than formal spatial immune exclusion, CD8-to-macrophage proximity, or multiplex co-localization. Sixth, CD163 and HO-1 support a heme-processing macrophage interpretation but do not by themselves prove a functional suppressive macrophage program. Seventh, the MSI-H/dMMR subgroup and the IHC subset were not powered for definitive MSI-stratified mechanistic conclusions. Finally, although HCIP was biologically motivated, it requires independent validation and refinement before clinical use.

In summary, fibrinogen appears to be the most practical single coagulation marker in this cohort, but HCIP offers a broader biological interpretation. By combining local hemorrhagic pathology with systemic coagulation activation, HCIP identifies a CD163/HO-1-enriched heme-processing and coagulation-associated immune pattern linked to postoperative recurrence risk. This phenotype may provide a useful framework for future studies examining how hemorrhage, coagulation, and macrophage biology jointly shape colorectal cancer progression.

## Conclusion

In this real-world cohort of patients with resected colorectal cancer, HCIP-poor status identified a recurrence-prone phenotype characterized by local hemorrhagic pathology, systemic coagulation activation, CD163/HO-1 macrophage enrichment, local fibrinogen/fibrin and tissue factor deposition, and reduced CD8/CD163 balance. Fibrinogen was the most stable single coagulation marker, whereas HCIP provided a clinically accessible composite framework linking hemorrhage, coagulation, and heme-processing macrophage-associated immune remodeling.

## Data Availability

The raw data supporting the conclusions of this article will be made available by the authors, without undue reservation.
