# Supplementary material for: Hemorrhage-coagulation immune phenotype is associated with CD163/HO-1-enriched heme-processing macrophage remodeling and predicts recurrence in colorectal cancer: a real-world retrospective cohort study
Source: Front Immunol. 2026 Jun 29;17:1868145. doi: 10.3389/fimmu.2026.1868145 (PMC13357416; doi:10.3389/fimmu.2026.1868145)
Supplement: Supplementary file 1 [file Table1.docx]

# Supplementary material

**Supplementary Table S1. Operational definition of HCIP**

| **Component/domain** | **Variable** | **Operational definition** | **Axis positive/classification rule** |
| --- | --- | --- | --- |
| Local hemorrhagic axis | H&E intratumoral hemorrhage score | Four-tier score 0-3; positive component if score >=2 | Local axis positive if H&E hemorrhage score >=2 or hemosiderin/Prussian blue score >=2 |
| Local hemorrhagic axis | Hemosiderin / Prussian blue evidence | Four-tier score 0-3; positive component if score >=2 | Local axis positive if H&E hemorrhage score >=2 or hemosiderin/Prussian blue score >=2 |
| Systemic coagulation axis | Fibrinogen | >4.0 g/L | Systemic axis positive if at least two of fibrinogen >4.0 g/L, D-dimer >1.0 mg/L, and platelet count >=300 x 10^9/L are present |
| Systemic coagulation axis | D-dimer | >1.0 mg/L | Systemic axis positive if at least two of fibrinogen >4.0 g/L, D-dimer >1.0 mg/L, and platelet count >=300 x 10^9/L are present |
| Systemic coagulation axis | Platelet count | >=300 x 10^9/L | Systemic axis positive if at least two of fibrinogen >4.0 g/L, D-dimer >1.0 mg/L, and platelet count >=300 x 10^9/L are present |
| HCIP classification | HCIP-favorable | Local axis negative and systemic coagulation axis negative | Reference phenotype |
| HCIP classification | HCIP-intermediate | Only one of the two axes positive | Intermediate phenotype |
| HCIP classification | HCIP-poor | Both local hemorrhagic axis and systemic coagulation axis positive | Poor phenotype |

*HCIP was constructed before outcome modeling from a local hemorrhagic axis and a systemic coagulation activation axis. The systemic coagulation axis is positive when at least two of fibrinogen >4.0 g/L, D-dimer >1.0 mg/L, and platelet count >=300 x 10^9/L are present. HCIP, hemorrhage-coagulation immune phenotype; H&E, hematoxylin and eosin.*

**Supplementary Table S2. Sensitivity analyses for alternative HCIP definitions**

| **Sensitivity definition** | **Comparison** | **HR (95% CI)** | **P value** | **N** | **Events** |
| --- | --- | --- | --- | --- | --- |
| Main definition | Intermediate | 1.36 (0.97-1.90) | 0.074 | 752 | 219 |
| Main definition | Poor | 1.91 (1.33-2.73) | <0.001 | 752 | 219 |
| Strict definition | Intermediate | 1.57 (1.16-2.12) | 0.003 | 752 | 219 |
| Strict definition | Poor | 1.53 (0.99-2.36) | 0.055 | 752 | 219 |
| H&E-only hemorrhage axis + fibrinogen/D-dimer axis | Intermediate | 1.43 (0.99-2.05) | 0.055 | 752 | 219 |
| H&E-only hemorrhage axis + fibrinogen/D-dimer axis | Poor | 1.54 (1.05-2.25) | 0.026 | 752 | 219 |
| Percentile-based composite definition | Intermediate | 1.11 (0.81-1.52) | 0.504 | 752 | 219 |
| Percentile-based composite definition | Poor | 1.69 (1.17-2.42) | 0.005 | 752 | 219 |
| Main definition after excluding antithrombotic users | Intermediate | 1.41 (1.00-2.01) | 0.053 | 707 | 205 |
| Main definition after excluding antithrombotic users | Poor | 1.98 (1.37-2.87) | <0.001 | 707 | 205 |

*Sensitivity analyses were adjusted using the same clinical covariate structure as the main HCIP model where estimable. HR, hazard ratio; CI, confidence interval.*

**Supplementary Table S3. Screening flow and cohort allocation**

| **Step** | **N** | **Percent of screened, %** |
| --- | --- | --- |
| Patients screened from radical colorectal adenocarcinoma surgery registry, January 2016-December 2025 | 1138 | 100.000 |
| Excluded: stage IV disease or non-radical surgery | 96 | 8.400 |
| Excluded: neoadjuvant treatment before index surgery | 114 | 10.000 |
| Excluded: synchronous malignancy or other ineligible history | 35 | 3.100 |
| Excluded: unavailable key hemorrhage/coagulation/pathology variables | 72 | 6.300 |
| Excluded: unavailable RFS or inadequate follow-up | 69 | 6.100 |
| Final analytic cohort | 752 | 66.100 |
| Model-development cohort | 525 | 46.100 |
| Validation cohort | 227 | 19.900 |
| Balanced FFPE/IHC subset | 90 | 7.900 |

*The exclusion counts sum to 386, leaving 752 patients in the final analytic cohort. The model-development and validation cohorts were generated using a stratified 7:3 split.*

**Supplementary Table S4. Follow-up and censoring profile**

| **HCIP group** | **N** | **Events** | **Censored** | **Observed follow-up median, months** | **Q1** | **Q3** | **Min** | **Max** | **Censored before 12 months** | **Censored before 36 months** | **Censored before 60 months** | **Reverse KM median follow-up** | **Lower 95% CI** | **Upper 95% CI** |
| --- | --- | --- | --- | --- | --- | --- | --- | --- | --- | --- | --- | --- | --- | --- |
| Favorable | 312 | 60 | 252 | 33.100 | 16.800 | 56.200 | 1.100 | 119.100 | 40 | 127 | 182 | 41.900 | 35.400 | 46.400 |
| Intermediate | 280 | 90 | 190 | 30.200 | 13.400 | 60.000 | 0.500 | 111.800 | 40 | 101 | 133 | 44.200 | 37.700 | 56.000 |
| Poor | 160 | 69 | 91 | 23.200 | 11.900 | 46.200 | 0.700 | 102.100 | 19 | 57 | 77 | 40.600 | 33.700 | 53.200 |
| Overall | 752 | 219 | 533 | 29.600 | 13.500 | 54.600 | 0.500 | 119.100 | 99 | 285 | 392 | 42.900 | 37.700 | 46.400 |

*Median follow-up was estimated using the reverse Kaplan-Meier method. Observed follow-up summaries use the recorded RFS follow-up time.*

**Supplementary Table S5. Overall missing-data profile**

| **Variable** | **Missing, n** | **Total N** | **Missing, %** |
| --- | --- | --- | --- |
| cd163_density | 512 | 752 | 68.100 |
| cd68_density | 512 | 752 | 68.100 |
| cd8_cd163_ratio | 512 | 752 | 68.100 |
| cd8_stromal_density | 512 | 752 | 68.100 |
| fibrinogen_ihc_hscore | 512 | 752 | 68.100 |
| ho1_hscore | 512 | 752 | 68.100 |
| macrophage_heme_score | 512 | 752 | 68.100 |
| tissue_factor_hscore | 512 | 752 | 68.100 |
| bmi | 19 | 752 | 2.500 |
| albumin_g_l | 14 | 752 | 1.900 |
| adjuvant_chemotherapy | 0 | 752 | 0.000 |
| age | 0 | 752 | 0.000 |
| ajcc_stage | 0 | 752 | 0.000 |
| anemia | 0 | 752 | 0.000 |
| cea_high | 0 | 752 | 0.000 |
| coagulation_axis_main | 0 | 752 | 0.000 |
| d_dimer_mg_l | 0 | 752 | 0.000 |
| fibrinogen_g_l | 0 | 752 | 0.000 |
| hcip | 0 | 752 | 0.000 |
| he_intratumoral_hemorrhage_score | 0 | 752 | 0.000 |
| hemoglobin_g_l | 0 | 752 | 0.000 |
| hemorrhage_axis_main | 0 | 752 | 0.000 |
| hemosiderin_score | 0 | 752 | 0.000 |
| histologic_grade | 0 | 752 | 0.000 |
| last_followup_date | 0 | 752 | 0.000 |
| lymphovascular_invasion | 0 | 752 | 0.000 |
| msi_status | 0 | 752 | 0.000 |
| nlr | 0 | 752 | 0.000 |
| patient_id | 0 | 752 | 0.000 |
| perineural_invasion | 0 | 752 | 0.000 |
| platelet_10e9_l | 0 | 752 | 0.000 |
| plr | 0 | 752 | 0.000 |
| radiotherapy | 0 | 752 | 0.000 |
| rfs_event | 0 | 752 | 0.000 |
| rfs_time_months | 0 | 752 | 0.000 |
| sex | 0 | 752 | 0.000 |
| sii | 0 | 752 | 0.000 |
| surgery_date | 0 | 752 | 0.000 |
| tumor_deposit | 0 | 752 | 0.000 |
| tumor_necrosis | 0 | 752 | 0.000 |
| tumor_site | 0 | 752 | 0.000 |
| tumor_size_cm | 0 | 752 | 0.000 |
| tumor_ulceration | 0 | 752 | 0.000 |

*IHC markers were intentionally measured only in the balanced FFPE subset; entries for IHC variables therefore indicate variables not assayed in the full cohort rather than random missingness. Clinical missingness was limited to BMI and albumin in this analysis set.*

**Supplementary Table S6. Missing-data profile according to HCIP group**

| **HCIP group** | **Group N** | **Variable** | **Missing, n** | **Missing, %** |
| --- | --- | --- | --- | --- |
| Favorable | 312 | adjuvant_chemotherapy | 0 | 0.000 |
| Intermediate | 280 | adjuvant_chemotherapy | 0 | 0.000 |
| Poor | 160 | adjuvant_chemotherapy | 0 | 0.000 |
| Favorable | 312 | age | 0 | 0.000 |
| Intermediate | 280 | age | 0 | 0.000 |
| Poor | 160 | age | 0 | 0.000 |
| Favorable | 312 | ajcc_stage | 0 | 0.000 |
| Intermediate | 280 | ajcc_stage | 0 | 0.000 |
| Poor | 160 | ajcc_stage | 0 | 0.000 |
| Favorable | 312 | albumin_g_l | 4 | 1.300 |
| Intermediate | 280 | albumin_g_l | 8 | 2.900 |
| Poor | 160 | albumin_g_l | 2 | 1.200 |
| Favorable | 312 | anemia | 0 | 0.000 |
| Intermediate | 280 | anemia | 0 | 0.000 |
| Poor | 160 | anemia | 0 | 0.000 |
| Favorable | 312 | bmi | 9 | 2.900 |
| Intermediate | 280 | bmi | 8 | 2.900 |
| Poor | 160 | bmi | 2 | 1.200 |
| Favorable | 312 | cd163_density | 225 | 72.100 |
| Intermediate | 280 | cd163_density | 198 | 70.700 |
| Poor | 160 | cd163_density | 89 | 55.600 |
| Favorable | 312 | cd68_density | 225 | 72.100 |
| Intermediate | 280 | cd68_density | 198 | 70.700 |
| Poor | 160 | cd68_density | 89 | 55.600 |
| Favorable | 312 | cd8_cd163_ratio | 225 | 72.100 |
| Intermediate | 280 | cd8_cd163_ratio | 198 | 70.700 |
| Poor | 160 | cd8_cd163_ratio | 89 | 55.600 |
| Favorable | 312 | cd8_stromal_density | 225 | 72.100 |
| Intermediate | 280 | cd8_stromal_density | 198 | 70.700 |
| Poor | 160 | cd8_stromal_density | 89 | 55.600 |
| Favorable | 312 | cea_high | 0 | 0.000 |
| Intermediate | 280 | cea_high | 0 | 0.000 |
| Poor | 160 | cea_high | 0 | 0.000 |
| Favorable | 312 | coagulation_axis_main | 0 | 0.000 |
| Intermediate | 280 | coagulation_axis_main | 0 | 0.000 |
| Poor | 160 | coagulation_axis_main | 0 | 0.000 |
| Favorable | 312 | d_dimer_mg_l | 0 | 0.000 |
| Intermediate | 280 | d_dimer_mg_l | 0 | 0.000 |
| Poor | 160 | d_dimer_mg_l | 0 | 0.000 |
| Favorable | 312 | fibrinogen_g_l | 0 | 0.000 |
| Intermediate | 280 | fibrinogen_g_l | 0 | 0.000 |
| Poor | 160 | fibrinogen_g_l | 0 | 0.000 |
| Favorable | 312 | fibrinogen_ihc_hscore | 225 | 72.100 |
| Intermediate | 280 | fibrinogen_ihc_hscore | 198 | 70.700 |
| Poor | 160 | fibrinogen_ihc_hscore | 89 | 55.600 |
| Favorable | 312 | he_intratumoral_hemorrhage_score | 0 | 0.000 |
| Intermediate | 280 | he_intratumoral_hemorrhage_score | 0 | 0.000 |
| Poor | 160 | he_intratumoral_hemorrhage_score | 0 | 0.000 |
| Favorable | 312 | hemoglobin_g_l | 0 | 0.000 |
| Intermediate | 280 | hemoglobin_g_l | 0 | 0.000 |
| Poor | 160 | hemoglobin_g_l | 0 | 0.000 |
| Favorable | 312 | hemorrhage_axis_main | 0 | 0.000 |
| Intermediate | 280 | hemorrhage_axis_main | 0 | 0.000 |
| Poor | 160 | hemorrhage_axis_main | 0 | 0.000 |
| Favorable | 312 | hemosiderin_score | 0 | 0.000 |
| Intermediate | 280 | hemosiderin_score | 0 | 0.000 |
| Poor | 160 | hemosiderin_score | 0 | 0.000 |
| Favorable | 312 | histologic_grade | 0 | 0.000 |
| Intermediate | 280 | histologic_grade | 0 | 0.000 |
| Poor | 160 | histologic_grade | 0 | 0.000 |
| Favorable | 312 | ho1_hscore | 225 | 72.100 |
| Intermediate | 280 | ho1_hscore | 198 | 70.700 |
| Poor | 160 | ho1_hscore | 89 | 55.600 |
| Favorable | 312 | last_followup_date | 0 | 0.000 |
| Intermediate | 280 | last_followup_date | 0 | 0.000 |
| Poor | 160 | last_followup_date | 0 | 0.000 |
| Favorable | 312 | lymphovascular_invasion | 0 | 0.000 |
| Intermediate | 280 | lymphovascular_invasion | 0 | 0.000 |
| Poor | 160 | lymphovascular_invasion | 0 | 0.000 |
| Favorable | 312 | macrophage_heme_score | 225 | 72.100 |
| Intermediate | 280 | macrophage_heme_score | 198 | 70.700 |
| Poor | 160 | macrophage_heme_score | 89 | 55.600 |
| Favorable | 312 | msi_status | 0 | 0.000 |
| Intermediate | 280 | msi_status | 0 | 0.000 |
| Poor | 160 | msi_status | 0 | 0.000 |
| Favorable | 312 | nlr | 0 | 0.000 |
| Intermediate | 280 | nlr | 0 | 0.000 |
| Poor | 160 | nlr | 0 | 0.000 |
| Favorable | 312 | patient_id | 0 | 0.000 |
| Intermediate | 280 | patient_id | 0 | 0.000 |
| Poor | 160 | patient_id | 0 | 0.000 |
| Favorable | 312 | perineural_invasion | 0 | 0.000 |
| Intermediate | 280 | perineural_invasion | 0 | 0.000 |
| Poor | 160 | perineural_invasion | 0 | 0.000 |
| Favorable | 312 | platelet_10e9_l | 0 | 0.000 |
| Intermediate | 280 | platelet_10e9_l | 0 | 0.000 |
| Poor | 160 | platelet_10e9_l | 0 | 0.000 |
| Favorable | 312 | plr | 0 | 0.000 |
| Intermediate | 280 | plr | 0 | 0.000 |
| Poor | 160 | plr | 0 | 0.000 |
| Favorable | 312 | radiotherapy | 0 | 0.000 |
| Intermediate | 280 | radiotherapy | 0 | 0.000 |
| Poor | 160 | radiotherapy | 0 | 0.000 |
| Favorable | 312 | rfs_event | 0 | 0.000 |
| Intermediate | 280 | rfs_event | 0 | 0.000 |
| Poor | 160 | rfs_event | 0 | 0.000 |
| Favorable | 312 | rfs_time_months | 0 | 0.000 |
| Intermediate | 280 | rfs_time_months | 0 | 0.000 |
| Poor | 160 | rfs_time_months | 0 | 0.000 |
| Favorable | 312 | sex | 0 | 0.000 |
| Intermediate | 280 | sex | 0 | 0.000 |
| Poor | 160 | sex | 0 | 0.000 |
| Favorable | 312 | sii | 0 | 0.000 |
| Intermediate | 280 | sii | 0 | 0.000 |
| Poor | 160 | sii | 0 | 0.000 |
| Favorable | 312 | surgery_date | 0 | 0.000 |
| Intermediate | 280 | surgery_date | 0 | 0.000 |
| Poor | 160 | surgery_date | 0 | 0.000 |
| Favorable | 312 | tissue_factor_hscore | 225 | 72.100 |
| Intermediate | 280 | tissue_factor_hscore | 198 | 70.700 |
| Poor | 160 | tissue_factor_hscore | 89 | 55.600 |
| Favorable | 312 | tumor_deposit | 0 | 0.000 |
| Intermediate | 280 | tumor_deposit | 0 | 0.000 |
| Poor | 160 | tumor_deposit | 0 | 0.000 |
| Favorable | 312 | tumor_necrosis | 0 | 0.000 |
| Intermediate | 280 | tumor_necrosis | 0 | 0.000 |
| Poor | 160 | tumor_necrosis | 0 | 0.000 |
| Favorable | 312 | tumor_site | 0 | 0.000 |
| Intermediate | 280 | tumor_site | 0 | 0.000 |
| Poor | 160 | tumor_site | 0 | 0.000 |
| Favorable | 312 | tumor_size_cm | 0 | 0.000 |
| Intermediate | 280 | tumor_size_cm | 0 | 0.000 |
| Poor | 160 | tumor_size_cm | 0 | 0.000 |
| Favorable | 312 | tumor_ulceration | 0 | 0.000 |
| Intermediate | 280 | tumor_ulceration | 0 | 0.000 |
| Poor | 160 | tumor_ulceration | 0 | 0.000 |

*This table describes missing values or variables not measured by design according to HCIP group.*

**Supplementary Table S7. Proportional hazards assumption checks**

| **Term** | **Chi-square** | **df** | **P value** |
| --- | --- | --- | --- |
| age | 0.248 | 1 | 0.619 |
| sex | 0.011 | 1 | 0.918 |
| bmi | 0.021 | 1 | 0.885 |
| tumor_site | 4.139 | 2 | 0.126 |
| ajcc_stage | 0.780 | 2 | 0.677 |
| histologic_grade | 0.438 | 2 | 0.803 |
| tumor_size_cm | 1.421 | 1 | 0.233 |
| lymphovascular_invasion | 0.034 | 1 | 0.854 |
| perineural_invasion | 0.015 | 1 | 0.901 |
| tumor_deposit | 0.000 | 1 | 0.998 |
| msi_status | 5.929 | 1 | 0.015 |
| tumor_ulceration | 0.941 | 1 | 0.332 |
| tumor_necrosis | 0.427 | 1 | 0.513 |
| albumin_g_l | 0.086 | 1 | 0.769 |
| cea_high | 0.417 | 1 | 0.518 |
| adjuvant_chemotherapy | 0.115 | 1 | 0.735 |
| radiotherapy | 0.378 | 1 | 0.539 |
| hcip | 0.842 | 2 | 0.656 |
| GLOBAL | 16.867 | 22 | 0.771 |

*Proportional hazards were evaluated using Schoenfeld residual-based tests. HCIP and the global model did not show evidence of violation; MSI/MMR status showed evidence of non-proportionality and was addressed using MSI-stratified sensitivity analysis.*

**Supplementary Table S8. Expanded restricted cubic spline checks for continuous predictors**

| **Variable** | **N** | **Events** | **Spline df** | **Reference median** | **P overall** | **P nonlinearity** | **Interpretation** |
| --- | --- | --- | --- | --- | --- | --- | --- |
| Fibrinogen (g/L) | 752 | 219 | 4 | 3.830 | <0.001 | 0.737 | No significant nonlinearity |
| D-dimer (mg/L) | 752 | 219 | 4 | 0.850 | 0.217 | 0.440 | No significant nonlinearity |
| Platelet count (x10^9/L) | 752 | 219 | 4 | 284.500 | 0.895 | 0.922 | No significant nonlinearity |
| SII | 752 | 219 | 4 | 820.000 | 0.239 | 0.894 | No significant nonlinearity |
| Age (years) | 752 | 219 | 4 | 62.000 | 0.895 | 0.810 | No significant nonlinearity |
| Tumor size (cm) | 752 | 219 | 4 | 4.900 | 0.943 | 0.896 | No significant nonlinearity |
| Albumin (g/L) | 738 | 217 | 4 | 39.600 | 0.899 | 0.805 | No significant nonlinearity |

*Restricted cubic spline models used 4 degrees of freedom and likelihood-ratio tests. No statistically significant nonlinearity was identified for the continuous predictors included in the functional-form assessment.*

**Supplementary Table S9. Stage II/III-only and T/N-stage-adjusted Cox sensitivity analyses**

| **Model** | **Comparison** | **HR (95% CI)** | **P value** | **N** | **Events** |
| --- | --- | --- | --- | --- | --- |
| Full cohort: AJCC-stage-adjusted model | HCIP intermediate vs favorable | 1.47 (1.03-2.10) | 0.033 | 719 | 209 |
| Full cohort: AJCC-stage-adjusted model | HCIP poor vs favorable | 2.07 (1.38-3.10) | <0.001 | 719 | 209 |
| Stage II/III-only: AJCC-stage-adjusted model | HCIP intermediate vs favorable | 1.41 (0.98-2.02) | 0.064 | 615 | 201 |
| Stage II/III-only: AJCC-stage-adjusted model | HCIP poor vs favorable | 1.95 (1.29-2.94) | 0.001 | 615 | 201 |
| Full cohort: T-stage and N-stage adjusted model | HCIP intermediate vs favorable | 1.51 (1.05-2.16) | 0.025 | 719 | 209 |
| Full cohort: T-stage and N-stage adjusted model | HCIP poor vs favorable | 2.09 (1.39-3.13) | <0.001 | 719 | 209 |
| Stage II/III-only: T-stage and N-stage adjusted model | HCIP intermediate vs favorable | 1.44 (1.00-2.07) | 0.052 | 615 | 201 |
| Stage II/III-only: T-stage and N-stage adjusted model | HCIP poor vs favorable | 1.93 (1.28-2.91) | 0.002 | 615 | 201 |

*Models used the same core clinical adjustment structure as the main Cox analysis, with either AJCC stage or separate T-stage and N-stage adjustment as indicated.*

**Supplementary Table S10. MSI/MMR-stratified sensitivity analyses**

| **Model** | **Comparison** | **HR (95% CI)** | **P value** | **N** | **Events** |
| --- | --- | --- | --- | --- | --- |
| Full cohort: Cox model stratified by MSI/MMR status | HCIP intermediate vs favorable | 1.46 (1.02-2.09) | 0.037 | 719 | 209 |
| Full cohort: Cox model stratified by MSI/MMR status | HCIP poor vs favorable | 2.00 (1.33-3.01) | <0.001 | 719 | 209 |
| Within MSI/MMR subgroup: MSS/pMMR | HCIP intermediate vs favorable | 1.36 (0.95-1.95) | 0.089 | 647 | 187 |
| Within MSI/MMR subgroup: MSS/pMMR | HCIP poor vs favorable | 1.78 (1.21-2.63) | 0.004 | 647 | 187 |
| Within MSI/MMR subgroup: MSI-H/dMMR | HCIP intermediate vs favorable | 1.41 (0.34-5.82) | 0.637 | 91 | 30 |
| Within MSI/MMR subgroup: MSI-H/dMMR | HCIP poor vs favorable | 5.03 (1.15-22.10) | 0.032 | 91 | 30 |

*The full-cohort sensitivity model was stratified by MSI/MMR status. Within-subgroup estimates are descriptive, particularly for MSI-H/dMMR cases because of limited subgroup size and event counts.*

**Supplementary Table S11. Recurrence-only endpoint sensitivity analysis**

| **Model** | **Comparison** | **HR (95% CI)** | **P value** | **N** | **Events** |
| --- | --- | --- | --- | --- | --- |
| Recurrence-only sensitivity model | HCIP intermediate vs favorable | 1.43 (1.00-2.04) | 0.050 | 719 | 209 |
| Recurrence-only sensitivity model | HCIP poor vs favorable | 2.06 (1.38-3.10) | <0.001 | 719 | 209 |

*Deaths without documented recurrence were treated as censoring events at death date when available; otherwise, censoring used last follow-up date.*

**Supplementary Table S12. Inverse-probability-weighted Cox sensitivity analyses**

| **Analysis** | **Comparison** | **HR (95% CI)** | **P value** |
| --- | --- | --- | --- |
| Stabilized multinomial IPTW; exposure = 3-level HCIP | HCIP poor vs intermediate/favorable reference | 1.40 (0.95-2.06) | 0.090 |
| Stabilized multinomial IPTW; exposure = 3-level HCIP | HCIP favorable vs intermediate/poor reference | 0.71 (0.50-1.03) | 0.069 |
| Stabilized binary IPTW; exposure = HCIP-poor vs non-poor | HCIP poor vs non-poor | 1.68 (1.19-2.39) | 0.003 |

*Stabilized inverse-probability weights were used. Weighted analyses were interpreted as supportive because some tumor-burden variables retained residual imbalance after weighting.*

**Supplementary Table S13. Covariate balance before and after IPTW**

| **Variable** | **SMD before weighting** | **SMD after IPTW** |
| --- | --- | --- |
| ajcc_stage | 0.659 | 0.193 |
| tumor_necrosis | 0.582 | 0.167 |
| tumor_ulceration | 0.568 | 0.179 |
| tumor_size_cm | 0.316 | 0.116 |
| albumin_g_l | 0.250 | 0.062 |
| tumor_site | 0.179 | 0.060 |
| alcohol_status | 0.175 | 0.074 |
| diabetes | 0.163 | 0.010 |
| histologic_grade | 0.138 | 0.087 |
| lymphovascular_invasion | 0.123 | 0.122 |
| sex | 0.112 | 0.085 |
| cea_high | 0.105 | 0.004 |
| hypertension | 0.087 | 0.051 |
| adjuvant_chemotherapy | 0.059 | 0.087 |
| age | 0.045 | 0.092 |
| radiotherapy | 0.041 | 0.000 |
| smoking_status | 0.038 | 0.079 |
| bmi | 0.023 | 0.029 |
| tumor_deposit | 0.023 | 0.029 |
| perineural_invasion | 0.018 | 0.009 |
| msi_status | 0.010 | 0.020 |

*Standardized mean differences are shown before and after IPTW. Values below approximately 0.10 are commonly considered acceptable, but selected tumor-burden variables remained partially imbalanced.*

**Supplementary Table S14. Ablation analysis of hemorrhage and coagulation feature sets**

| **Model** | **C_index** | **Delta_C_index** | **AUC_36m** | **Delta_AUC_36m** | **AUC_60m** | **Delta_AUC_60m** | **Brier_36m** | **Brier_60m** |
| --- | --- | --- | --- | --- | --- | --- | --- | --- |
| Clinical model | 0.676 | 0.000 | 0.756 | 0.000 | 0.743 | 0.000 | 0.276 | 0.326 |
| Clinical + local hemorrhagic axis | 0.675 | -0.001 | 0.753 | -0.003 | 0.741 | -0.002 | 0.267 | 0.315 |
| Clinical + systemic coagulation axis | 0.698 | 0.022 | 0.776 | 0.020 | 0.790 | 0.047 | 0.235 | 0.268 |
| Clinical + fibrinogen | 0.696 | 0.020 | 0.768 | 0.012 | 0.779 | 0.036 | 0.214 | 0.261 |
| Clinical + HCIP | 0.686 | 0.010 | 0.753 | -0.003 | 0.769 | 0.026 | 0.214 | 0.251 |
| Clinical + hemorrhage/coagulation components | 0.695 | 0.019 | 0.768 | 0.012 | 0.788 | 0.044 | 0.210 | 0.250 |

*Positive delta values for C-index and AUC indicate higher discrimination than the clinical model. Lower Brier scores indicate lower prediction error.*

**Supplementary Table S15. Bootstrap confidence intervals for incremental discrimination metrics**

| **Model** | **Metric** | **Estimate (95% bootstrap CI)** | **Interpretation** |
| --- | --- | --- | --- |
| Clinical + fibrinogen | Delta_C_index | 0.020 (-0.007, 0.044) | Positive values indicate higher discrimination than the clinical model. |
| Clinical + fibrinogen | Delta_AUC_36m | 0.012 (-0.014, 0.040) | Positive values indicate higher discrimination than the clinical model. |
| Clinical + fibrinogen | Delta_AUC_60m | 0.036 (-0.001, 0.074) | Positive values indicate higher discrimination than the clinical model. |
| Clinical + HCIP | Delta_C_index | 0.008 (-0.012, 0.029) | Positive values indicate higher discrimination than the clinical model. |
| Clinical + HCIP | Delta_AUC_36m | -0.004 (-0.033, 0.020) | Positive values indicate higher discrimination than the clinical model. |
| Clinical + HCIP | Delta_AUC_60m | 0.024 (-0.004, 0.053) | Positive values indicate higher discrimination than the clinical model. |
| Clinical + hemorrhage/coagulation components | Delta_C_index | 0.021 (-0.009, 0.053) | Positive values indicate higher discrimination than the clinical model. |
| Clinical + hemorrhage/coagulation components | Delta_AUC_36m | 0.012 (-0.019, 0.049) | Positive values indicate higher discrimination than the clinical model. |
| Clinical + hemorrhage/coagulation components | Delta_AUC_60m | 0.043 (-0.002, 0.086) | Positive values indicate higher discrimination than the clinical model. |

*Bootstrap confidence intervals are shown for discrimination deltas relative to the clinical Cox model. These intervals support conservative interpretation of the incremental discrimination gains.*

**Supplementary Table S16. Thirty-six-month recurrence prediction summary in the validation cohort**

| **Model** | **N_known_36m** | **Events_36m** | **AUC_36m_binary** | **Brier_36m_complete_case** | **Calibration_intercept** | **Calibration_slope** |
| --- | --- | --- | --- | --- | --- | --- |
| Clinical model | 143 | 49 | 0.755 | 0.262 | -1.131 | 0.571 |
| Clinical + local hemorrhagic axis | 143 | 49 | 0.751 | 0.254 | -1.073 | 0.584 |
| Clinical + systemic coagulation axis | 143 | 49 | 0.775 | 0.227 | -1.013 | 0.638 |
| Clinical + fibrinogen | 143 | 49 | 0.765 | 0.271 | 2.159 | 0.992 |
| Clinical + HCIP | 143 | 49 | 0.751 | 0.213 | -0.717 | 0.667 |
| Clinical + hemorrhage/coagulation components | 143 | 49 | 0.764 | 0.265 | 2.035 | 0.992 |

*This table summarizes complete-case binary prediction metrics at 36 months, complementing survival-model C-index, time-dependent AUC, Brier score, calibration, and decision-curve analyses.*

**Supplementary Table S17. Descriptive IHC features according to MSI/MMR status in the IHC subset**

| **Variable** | **MSS/pMMR** | **MSS/pMMR N** | **MSI-H/dMMR** | **MSI-H/dMMR N** |
| --- | --- | --- | --- | --- |
| cd8_stromal_density | 196.7 (161.8, 238.9) | 209 | 222.9 (180.9, 283.7) | 31 |
| cd68_density | 180.7 (157.2, 202.8) | 209 | 176.5 (149.6, 202.9) | 31 |
| cd163_density | 131.5 (102.2, 162.8) | 209 | 141.1 (118.1, 167.1) | 31 |
| ho1_hscore | 115.0 (92.8, 153.9) | 209 | 120.2 (99.3, 153.9) | 31 |
| fibrinogen_ihc_hscore | 103.0 (78.4, 126.0) | 209 | 92.1 (68.4, 111.8) | 31 |
| tissue_factor_hscore | 80.4 (61.1, 97.4) | 209 | 69.9 (56.6, 86.4) | 31 |
| cd8_cd163_ratio | 1.5 (1.1, 2.1) | 209 | 1.4 (1.2, 2.1) | 31 |
| macrophage_heme_score | -0.1 (-0.6, 0.6) | 209 | -0.1 (-0.6, 0.4) | 31 |

*Values are median (interquartile range). This descriptive table was not powered for formal inference within MSI/MMR strata.*

**Supplementary Figure S1. Restricted cubic spline analyses of continuous predictors.**


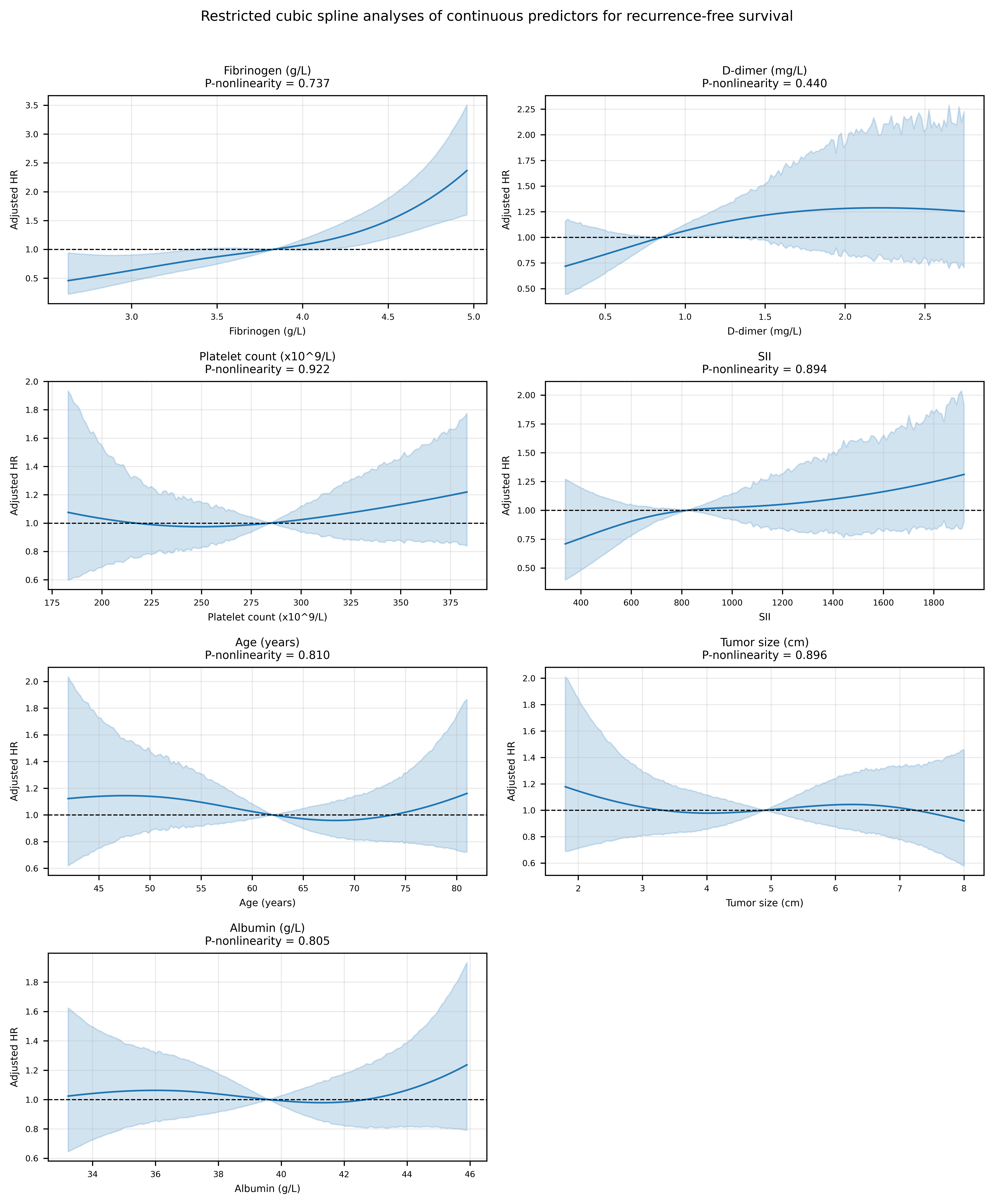


*Curves show adjusted hazard ratios across the 2.5th to 97.5th percentile range of each continuous predictor, referenced to the median value.*

**Supplementary Figure S2. Bootstrap confidence intervals for incremental validation metrics.**


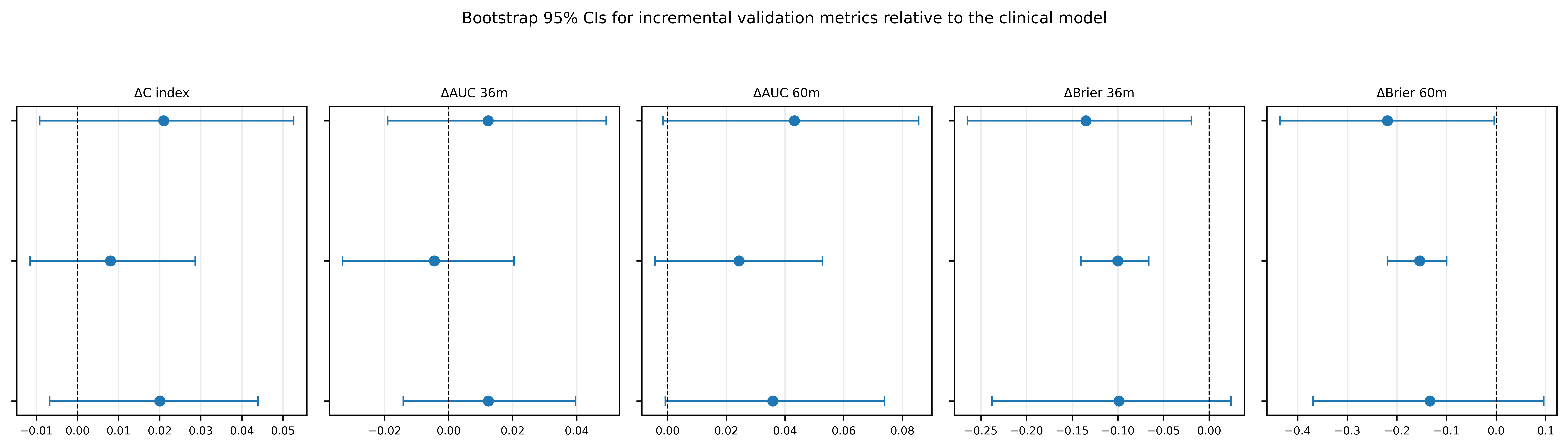


*Point estimates and 95% bootstrap intervals are shown relative to the clinical Cox model.*

**Supplementary Figure S3. Decision-curve analysis at 36 months.**


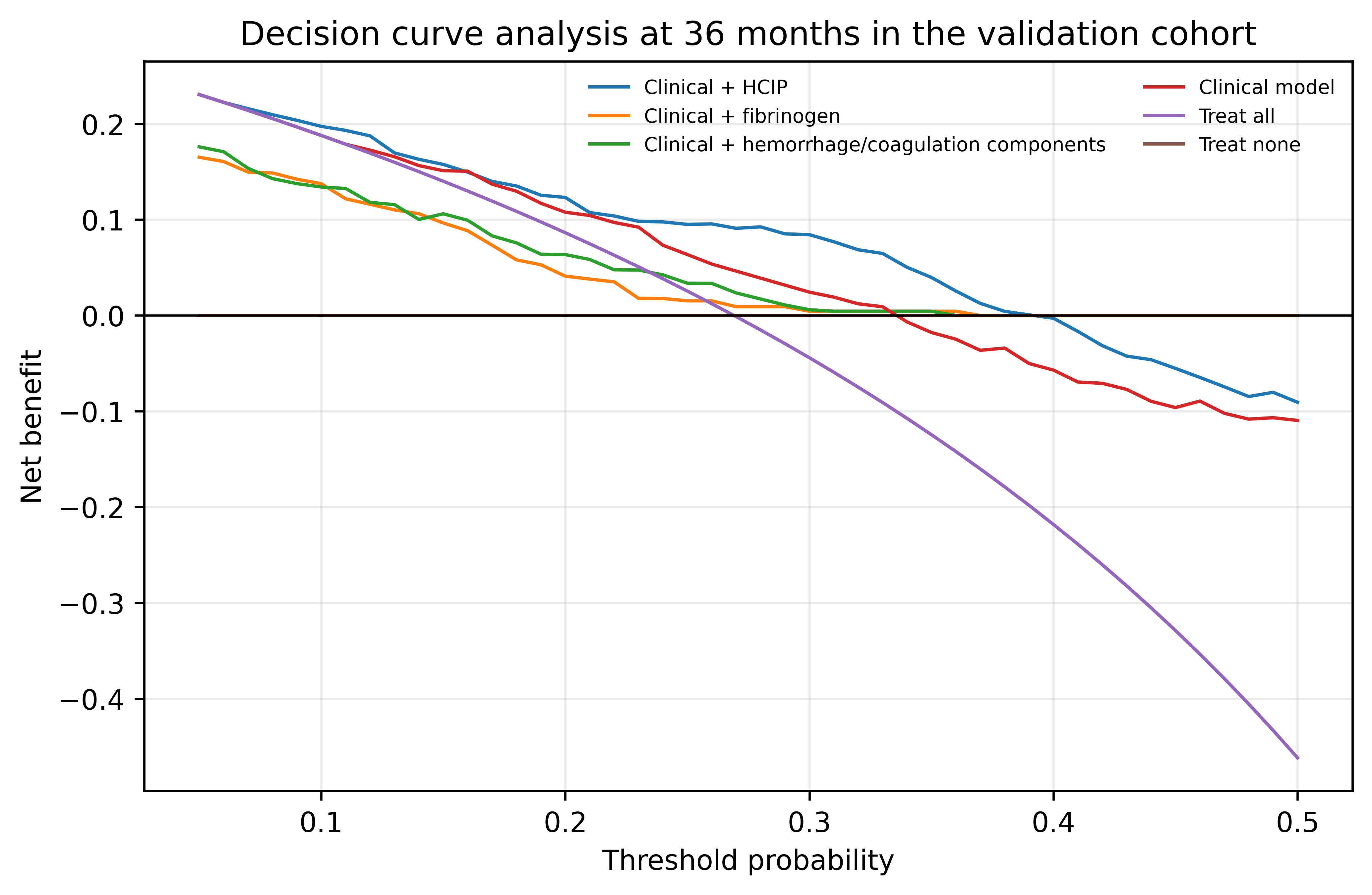


*Net benefit is shown across clinically plausible threshold probabilities in the validation cohort.*

**Supplementary Figure S4. Calibration by validation-cohort risk quintiles.**


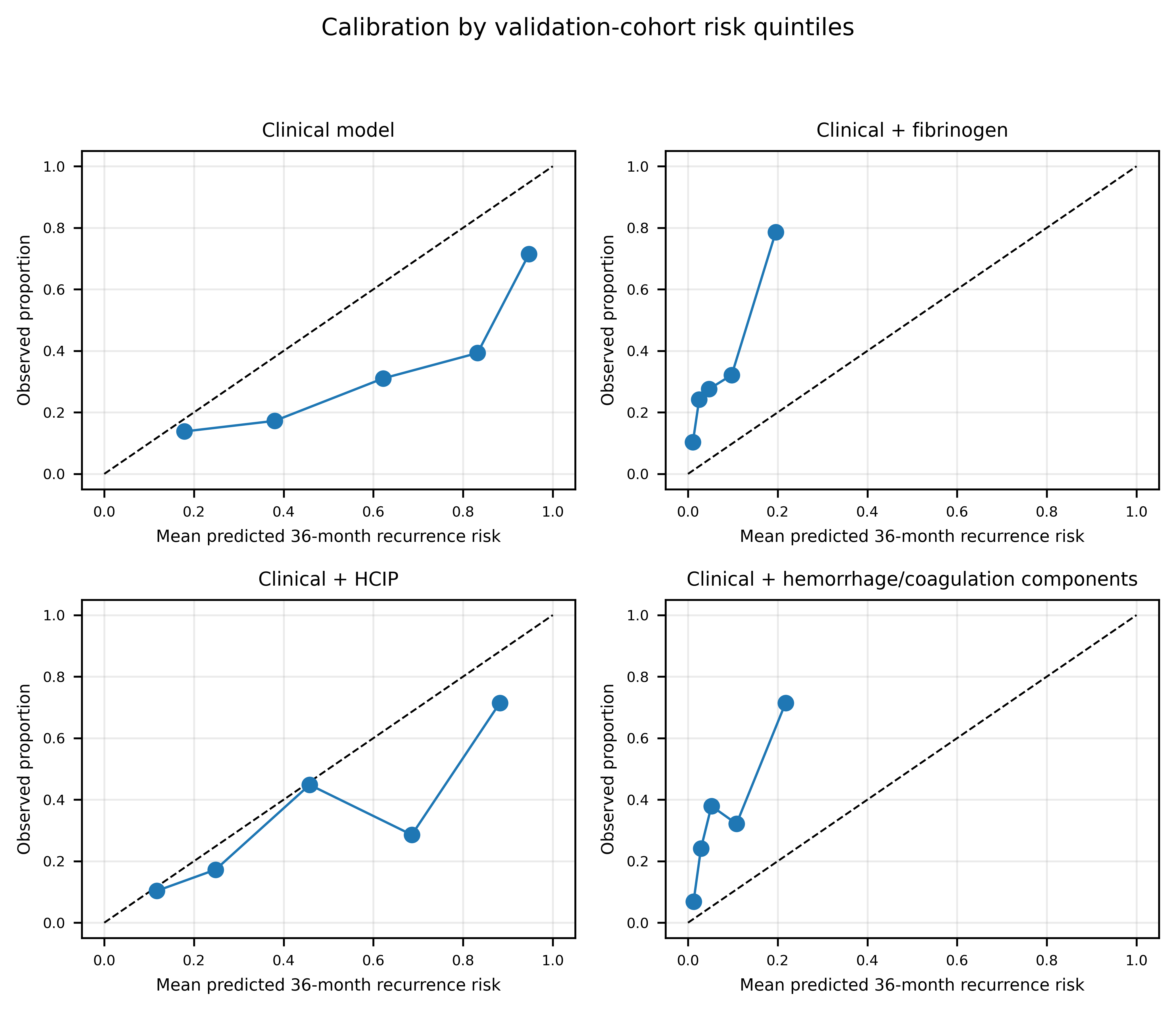


*Observed and predicted 36-month recurrence proportions are shown by risk quintiles.*
